# Supplementary material for: Increased BMI has a linear association with late-onset preeclampsia: A population-based study
Source: PLoS One. 2019 Oct 17;14(10):e0223888. doi: 10.1371/journal.pone.0223888 (PMC6797165; doi:10.1371/journal.pone.0223888)
Supplement: S4 File — (DOCX) [file pone.0223888.s004.docx]

**Logopen c:\aresult\eop vs lop 19.doc
EpiData Analysis V2.2.2.183 19/02/2019 11:30**

**. define risk #**

**Var Name risk of type Integer
Var length: 1decimals 0**

**. let risk = .**

**. if (preec = 1) and (terme <34) then risk = 1**

**. risk = 1**

**. if (preec = 1) and terme > 33 then risk = 0**

**. risk = 0**

**.. tables risk an /t /o /c /r**

**Select: (mult = 0)**

| **Outcome:risk** | | | | | | |
| --- | --- | --- | --- | --- | --- | --- |
| **an** | **0** | **%** | **1** | **%** | **Total** | **%** |
| **1** | **52** | **(65.0) {4.5}** | **28** | **(35.0) {4.9}** | **80** | **(100.0) {4.6}** |
| **2** | **36** | **(52.9) {3.1}** | **32** | **(47.1) {5.6}** | **68** | **(100.0) {3.9}** |
| **3** | **32** | **(50.8) {2.8}** | **31** | **(49.2) {5.4}** | **63** | **(100.0) {3.6}** |
| **4** | **44** | **(61.1) {3.8}** | **28** | **(38.9) {4.9}** | **72** | **(100.0) {4.1}** |
| **5** | **38** | **(52.8) {3.3}** | **34** | **(47.2) {5.9}** | **72** | **(100.0) {4.1}** |
| **6** | **60** | **(64.5) {5.2}** | **33** | **(35.5) {5.7}** | **93** | **(100.0) {5.4}** |
| **7** | **74** | **(69.2) {6.4}** | **33** | **(30.8) {5.7}** | **107** | **(100.0) {6.2}** |
| **8** | **94** | **(78.3) {8.1}** | **26** | **(21.7) {4.5}** | **120** | **(100.0) {6.9}** |
| **9** | **87** | **(76.3) {7.5}** | **27** | **(23.7) {4.7}** | **114** | **(100.0) {6.6}** |
| **10** | **82** | **(70.1) {7.1}** | **35** | **(29.9) {6.1}** | **117** | **(100.0) {6.7}** |
| **11** | **72** | **(67.3) {6.2}** | **35** | **(32.7) {6.1}** | **107** | **(100.0) {6.2}** |
| **12** | **54** | **(62.8) {4.6}** | **32** | **(37.2) {5.6}** | **86** | **(100.0) {5.0}** |
| **13** | **85** | **(71.4) {7.3}** | **34** | **(28.6) {5.9}** | **119** | **(100.0) {6.9}** |
| **14** | **61** | **(67.8) {5.2}** | **29** | **(32.2) {5.1}** | **90** | **(100.0) {5.2}** |
| **15** | **62** | **(68.1) {5.3}** | **29** | **(31.9) {5.1}** | **91** | **(100.0) {5.2}** |
| **16** | **68** | **(68.0) {5.9}** | **32** | **(32.0) {5.6}** | **100** | **(100.0) {5.8}** |
| **17** | **72** | **(66.1) {6.2}** | **37** | **(33.9) {6.4}** | **109** | **(100.0) {6.3}** |
| **18** | **89** | **(69.5) {7.7}** | **39** | **(30.5) {6.8}** | **128** | **(100.0) {7.4}** |
| **Total** | **1162** | **(66.9) {100.0}** | **574** | **(33.1) {100.0}** | **1736** |  |
| **Percents: (Row) {Col}     Chi^2^= 36.105 df(17) p= 0.0044** | | | | | | |

**. means age risk /t**

**Select: (mult = 0)**

**Syntax: Means AGE /BY= risk**

| **Age** | | | | | | | | | |
| --- | --- | --- | --- | --- | --- | --- | --- | --- | --- |
| **risk** | **Obs.** | **Sum** | **Mean** | **Variance** | **Std Dev** | **( 95% CI** | **mean )** | **Std Err** |  |
| **0** | **1162** | **33209.0** | **28.58** | **50.14** | **7.08** | **28.17** | **28.99** | **0.21** |  |
| **1** | **574** | **16940.0** | **29.51** | **47.30** | **6.88** | **28.95** | **30.08** | **0.29** |  |
|  |  |  |  |  |  |  |  |  |  |
| **risk** | **Minimum** | **p5** | **p10** | **p25** | **Median** | **p75** | **p90** | **p95** | **Max** |
| **0** | **14.00** | **18.00** | **19.00** | **23.00** | **28.00** | **34.00** | **38.00** | **40.85** | **47.00** |
| **1** | **14.00** | **18.00** | **20.00** | **24.00** | **30.00** | **35.00** | **39.00** | **41.00** | **46.00** |

| **Source** | **SS** | **df** | **MS** | **F** | **p Value** |
| --- | --- | --- | --- | --- | --- |
| **Between** | **334.47** | **1** | **334.47** | **6.80** | **0.0092** |
| **Within** | **85316.63** | **1734** | **49.20** |  |  |
| **Total** | **85651.10** | **1735** | **49.37** |  |  |
| **Bartlett's test for homogeneity of variance  Chi^2^= 0.645 df(1) p= 0.422** | | | | | |

**. tables risk ageg /t /o /c /r**

**Select: (mult = 0)**

| **Outcome:risk** | | | | | | |
| --- | --- | --- | --- | --- | --- | --- |
| **ageg** | **0** | **%** | **1** | **%** | **Total** | **%** |
| **10 - 14** | **3** | **(75.0) {0.3}** | **1** | **(25.0) {0.2}** | **4** | **(100.0) {0.2}** |
| **15 - 19** | **120** | **(72.7) {10.3}** | **45** | **(27.3) {7.8}** | **165** | **(100.0) {9.5}** |
| **20 - 24** | **258** | **(71.3) {22.2}** | **104** | **(28.7) {18.1}** | **362** | **(100.0) {20.9}** |
| **25 - 29** | **266** | **(67.7) {22.9}** | **127** | **(32.3) {22.1}** | **393** | **(100.0) {22.6}** |
| **30 - 34** | **238** | **(61.8) {20.5}** | **147** | **(38.2) {25.6}** | **385** | **(100.0) {22.2}** |
| **35 - 39** | **193** | **(64.3) {16.6}** | **107** | **(35.7) {18.6}** | **300** | **(100.0) {17.3}** |
| **40 - 44** | **81** | **(65.9) {7.0}** | **42** | **(34.1) {7.3}** | **123** | **(100.0) {7.1}** |
| **45 - 49** | **3** | **(75.0) {0.3}** | **1** | **(25.0) {0.2}** | **4** | **(100.0) {0.2}** |
| **Total** | **1162** | **(66.9) {100.0}** | **574** | **(33.1) {100.0}** | **1736** |  |
| **Percents: (Row) {Col}     Chi^2^= 11.448 df(7) p= 0.1202    Cells_expected<5:_ 4 (25 pct.)** | | | | | | |

**. tables risk ado /t /o /c /r**

**Select: (mult = 0)**

| **Outcome:risk** | | | | | | |
| --- | --- | --- | --- | --- | --- | --- |
| **ado** | **1** | **%** | **0** | **%** | **Total** | **%** |
| **1** | **18** | **(30.5) {3.1}** | **41** | **(69.5) {3.5}** | **59** | **(100.0) {3.4}** |
| **0** | **556** | **(33.2) {96.9}** | **1121** | **(66.8) {96.5}** | **1677** | **(100.0) {96.6}** |
| **Total** | **574** | **(33.1) {100.0}** | **1162** | **(66.9) {100.0}** | **1736** |  |
| **Percents: (Row) {Col}  Exposure: ado = 1 Outcome: risk = 1    Chi^2^= 0.180 df(1) p= 0.6711    Odds Ratio = 0.89 (95% CI: 0.50-1.55)    (Robins,Greenland,Breslow CI)** | | | | | | |

**. tables risk a35 /t /o /c /r**

**Select: (mult = 0)**

| **Outcome:risk** | | | | | | |
| --- | --- | --- | --- | --- | --- | --- |
| **a35** | **1** | **%** | **0** | **%** | **Total** | **%** |
| **1** | **150** | **(35.1) {26.1}** | **277** | **(64.9) {23.8}** | **427** | **(100.0) {24.6}** |
| **0** | **424** | **(32.4) {73.9}** | **885** | **(67.6) {76.2}** | **1309** | **(100.0) {75.4}** |
| **Total** | **574** | **(33.1) {100.0}** | **1162** | **(66.9) {100.0}** | **1736** |  |
| **Percents: (Row) {Col}  Exposure: a35 = 1 Outcome: risk = 1    Chi^2^= 1.090 df(1) p= 0.2964    Odds Ratio = 1.13 (95% CI: 0.90-1.42)    (Robins,Greenland,Breslow CI)** | | | | | | |

**. means gest risk /t**

**Select: (mult = 0)**

**Syntax: Means GEST /BY= risk**

| **Gestit‚** | | | | | | | | | |
| --- | --- | --- | --- | --- | --- | --- | --- | --- | --- |
| **risk** | **Obs.** | **Sum** | **Mean** | **Variance** | **Std Dev** | **( 95% CI** | **mean )** | **Std Err** |  |
| **0** | **1162** | **3177.00** | **2.73** | **4.40** | **2.10** | **2.61** | **2.85** | **0.06** |  |
| **1** | **574** | **1671.00** | **2.91** | **4.24** | **2.06** | **2.74** | **3.08** | **0.09** |  |
|  |  |  |  |  |  |  |  |  |  |
| **risk** | **Minimum** | **p5** | **p10** | **p25** | **Median** | **p75** | **p90** | **p95** | **Max** |
| **0** | **1.0** | **1.0** | **1.0** | **1.0** | **2.00** | **4.00** | **6.00** | **7.00** | **14.00** |
| **1** | **1.0** | **1.0** | **1.0** | **1.0** | **2.00** | **4.00** | **6.00** | **7.00** | **13.00** |

| **Source** | **SS** | **df** | **MS** | **F** | **p Value** |
| --- | --- | --- | --- | --- | --- |
| **Between** | **12.05** | **1** | **12.05** | **2.77** | **0.0961** |
| **Within** | **7537.30** | **1734** | **4.35** |  |  |
| **Total** | **7549.35** | **1735** | **4.35** |  |  |
| **Bartlett's test for homogeneity of variance  Chi^2^= 0.252 df(1) p= 0.616** | | | | | |

**. tables risk g1 /t /o /c /r**

**Select: (mult = 0)**

| **Outcome:risk** | | | | | | |
| --- | --- | --- | --- | --- | --- | --- |
| **g1** | **1** | **%** | **0** | **%** | **Total** | **%** |
| **1** | **180** | **(29.5) {31.4}** | **430** | **(70.5) {37.0}** | **610** | **(100.0) {35.1}** |
| **0** | **394** | **(35.0) {68.6}** | **732** | **(65.0) {63.0}** | **1126** | **(100.0) {64.9}** |
| **Total** | **574** | **(33.1) {100.0}** | **1162** | **(66.9) {100.0}** | **1736** |  |
| **Percents: (Row) {Col}  Exposure: g1 = 1 Outcome: risk = 1    Chi^2^= 5.374 df(1) p= 0.0204    Odds Ratio = 0.78 (95% CI: 0.63-0.96)    (Robins,Greenland,Breslow CI)** | | | | | | |

**. means par risk /t**

**Select: (mult = 0)**

**Syntax: Means PAR /BY= risk**

| **Parit‚** | | | | | | | | | |
| --- | --- | --- | --- | --- | --- | --- | --- | --- | --- |
| **risk** | **Obs.** | **Sum** | **Mean** | **Variance** | **Std Dev** | **( 95% CI** | **mean )** | **Std Err** |  |
| **0** | **1162** | **1354.00** | **1.17** | **2.67** | **1.63** | **1.07** | **1.26** | **0.05** |  |
| **1** | **574** | **738.00** | **1.29** | **2.98** | **1.73** | **1.14** | **1.43** | **0.07** |  |
|  |  |  |  |  |  |  |  |  |  |
| **risk** | **Minimum** | **p5** | **p10** | **p25** | **Median** | **p75** | **p90** | **p95** | **Max** |
| **0** | **0.0** | **0.0** | **0.0** | **0.0** | **1.0** | **2.00** | **3.00** | **5.00** | **11.00** |
| **1** | **0.0** | **0.0** | **0.0** | **0.0** | **1.0** | **2.00** | **4.00** | **5.00** | **10.00** |

| **Source** | **SS** | **df** | **MS** | **F** | **p Value** |
| --- | --- | --- | --- | --- | --- |
| **Between** | **5.58** | **1** | **5.58** | **2.01** | **0.156** |
| **Within** | **4799.42** | **1734** | **2.77** |  |  |
| **Total** | **4805.00** | **1735** | **2.77** |  |  |
| **Bartlett's test for homogeneity of variance  Chi^2^= 2.358 df(1) p= 0.125** | | | | | |

**. tables risk p1 /t /o /c /r**

**Select: (mult = 0)**

| **Outcome:risk** | | | | | | |
| --- | --- | --- | --- | --- | --- | --- |
| **p1** | **1** | **%** | **0** | **%** | **Total** | **%** |
| **1** | **260** | **(31.1) {45.3}** | **577** | **(68.9) {49.7}** | **837** | **(100.0) {48.2}** |
| **0** | **314** | **(34.9) {54.7}** | **585** | **(65.1) {50.3}** | **899** | **(100.0) {51.8}** |
| **Total** | **574** | **(33.1) {100.0}** | **1162** | **(66.9) {100.0}** | **1736** |  |
| **Percents: (Row) {Col}  Exposure: p1 = 1 Outcome: risk = 1    Chi^2^= 2.925 df(1) p= 0.0872    Odds Ratio = 0.84 (95% CI: 0.69-1.03)    (Robins,Greenland,Breslow CI)** | | | | | | |

**. tables risk p5 /t /o /c /r**

**Select: (mult = 0)**

| **Outcome:risk** | | | | | | |
| --- | --- | --- | --- | --- | --- | --- |
| **p5** | **1** | **%** | **0** | **%** | **Total** | **%** |
| **1** | **63** | **(36.6) {11.0}** | **109** | **(63.4) {9.4}** | **172** | **(100.0) {9.9}** |
| **0** | **511** | **(32.7) {89.0}** | **1053** | **(67.3) {90.6}** | **1564** | **(100.0) {90.1}** |
| **Total** | **574** | **(33.1) {100.0}** | **1162** | **(66.9) {100.0}** | **1736** |  |
| **Percents: (Row) {Col}  Exposure: p5 = 1 Outcome: risk = 1    Chi^2^= 1.095 df(1) p= 0.2953    Odds Ratio = 1.19 (95% CI: 0.86-1.65)    (Robins,Greenland,Breslow CI)** | | | | | | |

**. tables risk sitfam /t /o /c /r**

**Select: (mult = 0)**

| **Outcome:risk** | | | | | | |
| --- | --- | --- | --- | --- | --- | --- |
| **Situation de famille** | **0** | **%** | **1** | **%** | **Total** | **%** |
| **CELIBAT** | **415** | **(68.5) {35.7}** | **191** | **(31.5) {33.3}** | **606** | **(100.0) {34.9}** |
| **CONCUBINE** | **428** | **(66.6) {36.9}** | **215** | **(33.4) {37.5}** | **643** | **(100.0) {37.1}** |
| **MARIEE** | **289** | **(64.8) {24.9}** | **157** | **(35.2) {27.4}** | **446** | **(100.0) {25.7}** |
| **DIV/SEPAREE** | **24** | **(70.6) {2.1}** | **10** | **(29.4) {1.7}** | **34** | **(100.0) {2.0}** |
| **VEUVE** | **5** | **(83.3) {0.4}** | **1** | **(16.7) {0.2}** | **6** | **(100.0) {0.3}** |
| **Total** | **1161** | **(66.9) {100.0}** | **574** | **(33.1) {100.0}** | **1735** |  |
| **Percents: (Row) {Col}     Chi^2^= 2.549 df(4) p= 0.6360    Cells_expected<5:_ 2 (20 pct.)** | | | | | | |

**. tables risk single /t /o /c /r**

**Select: (mult = 0)**

| **Outcome:risk** | | | | | | |
| --- | --- | --- | --- | --- | --- | --- |
| **single** | **1** | **%** | **0** | **%** | **Total** | **%** |
| **1** | **202** | **(31.3) {35.2}** | **444** | **(68.7) {38.2}** | **646** | **(100.0) {37.2}** |
| **0** | **372** | **(34.2) {64.8}** | **717** | **(65.8) {61.8}** | **1089** | **(100.0) {62.8}** |
| **Total** | **574** | **(33.1) {100.0}** | **1161** | **(66.9) {100.0}** | **1735** |  |
| **Percents: (Row) {Col}  Exposure: single = 1 Outcome: risk = 1    Chi^2^= 1.530 df(1) p= 0.2161    Odds Ratio = 0.88 (95% CI: 0.71-1.08)    (Robins,Greenland,Breslow CI)** | | | | | | |

**. tables risk origine /t /o /c /r**

**Select: (mult = 0)**

| **Outcome:risk** | | | | | | |
| --- | --- | --- | --- | --- | --- | --- |
| **Origine** | **0** | **%** | **1** | **%** | **Total** | **%** |
| **REUNION** | **967** | **(66.7) {83.4}** | **482** | **(33.3) {84.0}** | **1449** | **(100.0) {83.6}** |
| **MAYOTTE** | **77** | **(64.7) {6.6}** | **42** | **(35.3) {7.3}** | **119** | **(100.0) {6.9}** |
| **COMORES** | **10** | **(62.5) {0.9}** | **6** | **(37.5) {1.0}** | **16** | **(100.0) {0.9}** |
| **MADAGASCAR** | **22** | **(71.0) {1.9}** | **9** | **(29.0) {1.6}** | **31** | **(100.0) {1.8}** |
| **METROPOLE** | **67** | **(73.6) {5.8}** | **24** | **(26.4) {4.2}** | **91** | **(100.0) {5.2}** |
| **MAURICE** | **8** | **(57.1) {0.7}** | **6** | **(42.9) {1.0}** | **14** | **(100.0) {0.8}** |
| **AUTRE** | **9** | **(64.3) {0.8}** | **5** | **(35.7) {0.9}** | **14** | **(100.0) {0.8}** |
| **Total** | **1160** | **(66.9) {100.0}** | **574** | **(33.1) {100.0}** | **1734** |  |
| **Percents: (Row) {Col}     Chi^2^= 3.152 df(6) p= 0.7895    Cells_expected<5:_ 2 (14 pct.)** | | | | | | |

**. tables risk etude /t /o /c /r**

**Select: (mult = 0)**

| **Outcome:risk** | | | | | | |
| --- | --- | --- | --- | --- | --- | --- |
| **Niveau d'etudes** | **0** | **%** | **1** | **%** | **Total** | **%** |
| **AUCUNE** | **12** | **(44.4) {1.0}** | **15** | **(55.6) {2.6}** | **27** | **(100.0) {1.6}** |
| **PRIMAIRE** | **44** | **(72.1) {3.8}** | **17** | **(27.9) {3.0}** | **61** | **(100.0) {3.5}** |
| **COLLEGE** | **413** | **(68.8) {35.7}** | **187** | **(31.2) {32.6}** | **600** | **(100.0) {34.7}** |
| **TECHNIQUE** | **33** | **(73.3) {2.9}** | **12** | **(26.7) {2.1}** | **45** | **(100.0) {2.6}** |
| **LYCEE** | **358** | **(69.5) {30.9}** | **157** | **(30.5) {27.4}** | **515** | **(100.0) {29.8}** |
| **UNIVERSITE** | **258** | **(65.2) {22.3}** | **138** | **(34.8) {24.1}** | **396** | **(100.0) {22.9}** |
| **INCONNU** | **39** | **(45.3) {3.4}** | **47** | **(54.7) {8.2}** | **86** | **(100.0) {5.0}** |
| **Total** | **1157** | **(66.9) {100.0}** | **573** | **(33.1) {100.0}** | **1730** |  |
| **Percents: (Row) {Col}     Chi^2^= 28.921 df(6) p= 0.0001** | | | | | | |

**. tables risk study /t /o /c /r**

**Select: (mult = 0)**

| **Outcome:risk** | | | | | | |
| --- | --- | --- | --- | --- | --- | --- |
| **study** | **1** | **%** | **0** | **%** | **Total** | **%** |
| **1** | **295** | **(32.4) {56.1}** | **616** | **(67.6) {55.1}** | **911** | **(100.0) {55.4}** |
| **0** | **231** | **(31.5) {43.9}** | **502** | **(68.5) {44.9}** | **733** | **(100.0) {44.6}** |
| **Total** | **526** | **(32.0) {100.0}** | **1118** | **(68.0) {100.0}** | **1644** |  |
| **Percents: (Row) {Col}  Exposure: study = 1 Outcome: risk = 1    Chi^2^= 0.141 df(1) p= 0.7077    Odds Ratio = 1.04 (95% CI: 0.84-1.28)    (Robins,Greenland,Breslow CI)** | | | | | | |

**. tables risk prof /t /o /c /r**

**Select: (mult = 0)**

| **Outcome:risk** | | | | | | |
| --- | --- | --- | --- | --- | --- | --- |
| **Profession** | **0** | **%** | **1** | **%** | **Total** | **%** |
| **SANS** | **686** | **(68.7) {68.8}** | **312** | **(31.3) {68.6}** | **998** | **(100.0) {68.7}** |
| **AGRICULTEUR** | **4** | **(80.0) {0.4}** | **1** | **(20.0) {0.2}** | **5** | **(100.0) {0.3}** |
| **ARTISAN/COMM** | **15** | **(78.9) {1.5}** | **4** | **(21.1) {0.9}** | **19** | **(100.0) {1.3}** |
| **CADRES/SUP** | **13** | **(48.1) {1.3}** | **14** | **(51.9) {3.1}** | **27** | **(100.0) {1.9}** |
| **INTERMEDIAIRE** | **30** | **(71.4) {3.0}** | **12** | **(28.6) {2.6}** | **42** | **(100.0) {2.9}** |
| **EMPLOYES** | **156** | **(70.3) {15.6}** | **66** | **(29.7) {14.5}** | **222** | **(100.0) {15.3}** |
| **OUVRIERS** | **35** | **(71.4) {3.5}** | **14** | **(28.6) {3.1}** | **49** | **(100.0) {3.4}** |
| **ENSEIGNANTE** | **24** | **(70.6) {2.4}** | **10** | **(29.4) {2.2}** | **34** | **(100.0) {2.3}** |
| **MEDICAL/PARAMED** | **29** | **(56.9) {2.9}** | **22** | **(43.1) {4.8}** | **51** | **(100.0) {3.5}** |
| **PAS/D'INFO** | **5** | **(100.0) {0.5}** | **0** | **(0.0) {0.0}** | **5** | **(100.0) {0.3}** |
| **Total** | **997** | **(68.7) {100.0}** | **455** | **(31.3) {100.0}** | **1452** |  |
| **Percents: (Row) {Col}     Chi^2^= 12.747 df(9) p= 0.1744    Cells_expected<5:_ 4 (20 pct.)** | | | | | | |

**. tables risk tabac /t /o /c /r**

**Select: (mult = 0)**

| **Outcome:risk** | | | | | | |
| --- | --- | --- | --- | --- | --- | --- |
| **Tabac** | **1** | **%** | **0** | **%** | **Total** | **%** |
| **1** | **55** | **(35.5) {9.6}** | **100** | **(64.5) {8.6}** | **155** | **(100.0) {8.9}** |
| **0** | **519** | **(32.8) {90.4}** | **1061** | **(67.2) {91.4}** | **1580** | **(100.0) {91.1}** |
| **Total** | **574** | **(33.1) {100.0}** | **1161** | **(66.9) {100.0}** | **1735** |  |
| **Percents: (Row) {Col}  Exposure: Tabac = 1 Outcome: risk = 1    Chi^2^= 0.443 df(1) p= 0.5057    Odds Ratio = 1.12 (95% CI: 0.80-1.59)    (Robins,Greenland,Breslow CI)** | | | | | | |

**. tables risk alcool /t /o /c /r**

**Select: (mult = 0)**

| **Outcome:risk** | | | | | | |
| --- | --- | --- | --- | --- | --- | --- |
| **alcool** | **1** | **%** | **0** | **%** | **Total** | **%** |
| **1** | **3** | **(30.0) {0.5}** | **7** | **(70.0) {0.6}** | **10** | **(100.0) {0.6}** |
| **0** | **570** | **(33.1) {99.5}** | **1154** | **(66.9) {99.4}** | **1724** | **(100.0) {99.4}** |
| **Total** | **573** | **(33.0) {100.0}** | **1161** | **(67.0) {100.0}** | **1734** |  |
| **Percents: (Row) {Col}  Exposure: alcool = 1 Outcome: risk = 1    Chi^2^= 0.042 df(1) p= 0.8373    Cells_expected<5:_ 1 (25 pct.)   Odds Ratio = 0.87 (95% CI: 0.22-3.37)    (Robins,Greenland,Breslow CI)** | | | | | | |

**. tables risk med /t /o /c /r**

**Select: (mult = 0)**

| **Outcome:risk** | | | | | | |
| --- | --- | --- | --- | --- | --- | --- |
| **m Pathologie medicale** | **0** | **%** | **1** | **%** | **Total** | **%** |
| **0** | **810** | **(67.7) {69.8}** | **387** | **(32.3) {67.7}** | **1197** | **(100.0) {69.1}** |
| **1** | **64** | **(64.6) {5.5}** | **35** | **(35.4) {6.1}** | **99** | **(100.0) {5.7}** |
| **2** | **20** | **(83.3) {1.7}** | **4** | **(16.7) {0.7}** | **24** | **(100.0) {1.4}** |
| **3** | **8** | **(61.5) {0.7}** | **5** | **(38.5) {0.9}** | **13** | **(100.0) {0.8}** |
| **4** | **21** | **(67.7) {1.8}** | **10** | **(32.3) {1.7}** | **31** | **(100.0) {1.8}** |
| **5** | **1** | **(33.3) {0.1}** | **2** | **(66.7) {0.3}** | **3** | **(100.0) {0.2}** |
| **6** | **53** | **(58.9) {4.6}** | **37** | **(41.1) {6.5}** | **90** | **(100.0) {5.2}** |
| **7** | **27** | **(84.4) {2.3}** | **5** | **(15.6) {0.9}** | **32** | **(100.0) {1.8}** |
| **8** | **1** | **(25.0) {0.1}** | **3** | **(75.0) {0.5}** | **4** | **(100.0) {0.2}** |
| **9** | **7** | **(58.3) {0.6}** | **5** | **(41.7) {0.9}** | **12** | **(100.0) {0.7}** |
| **GOITRE** | **2** | **(66.7) {0.2}** | **1** | **(33.3) {0.2}** | **3** | **(100.0) {0.2}** |
| **HYPOTHYR** | **8** | **(61.5) {0.7}** | **5** | **(38.5) {0.9}** | **13** | **(100.0) {0.8}** |
| **HYPERTHYR** | **3** | **(60.0) {0.3}** | **2** | **(40.0) {0.3}** | **5** | **(100.0) {0.3}** |
| **DEF/PROTEINE/S** | **0** | **(0.0) {0.0}** | **2** | **(100.0) {0.3}** | **2** | **(100.0) {0.1}** |
| **PSYCHOSE** | **0** | **(0.0) {0.0}** | **2** | **(100.0) {0.3}** | **2** | **(100.0) {0.1}** |
| **MYOPATHIE** | **1** | **(33.3) {0.1}** | **2** | **(66.7) {0.3}** | **3** | **(100.0) {0.2}** |
| **CECITE** | **1** | **(100.0) {0.1}** | **0** | **(0.0) {0.0}** | **1** | **(100.0) {0.1}** |
| **SURDITE** | **4** | **(40.0) {0.3}** | **6** | **(60.0) {1.0}** | **10** | **(100.0) {0.6}** |
| **SPASMOPHILIE** | **6** | **(100.0) {0.5}** | **0** | **(0.0) {0.0}** | **6** | **(100.0) {0.3}** |
| **COAGULOPATHIE** | **6** | **(60.0) {0.5}** | **4** | **(40.0) {0.7}** | **10** | **(100.0) {0.6}** |
| **SCOLIOSE** | **3** | **(100.0) {0.3}** | **0** | **(0.0) {0.0}** | **3** | **(100.0) {0.2}** |
| **PSORIASIS** | **1** | **(100.0) {0.1}** | **0** | **(0.0) {0.0}** | **1** | **(100.0) {0.1}** |
| **PATHO/RENALE** | **10** | **(52.6) {0.9}** | **9** | **(47.4) {1.6}** | **19** | **(100.0) {1.1}** |
| **RAA** | **2** | **(100.0) {0.2}** | **0** | **(0.0) {0.0}** | **2** | **(100.0) {0.1}** |
| **POLIO** | **0** | **(0.0) {0.0}** | **1** | **(100.0) {0.2}** | **1** | **(100.0) {0.1}** |
| **NODULE/THYR** | **0** | **(0.0) {0.0}** | **1** | **(100.0) {0.2}** | **1** | **(100.0) {0.1}** |
| **MIGRAINE** | **6** | **(85.7) {0.5}** | **1** | **(14.3) {0.2}** | **7** | **(100.0) {0.4}** |
| **ECZEMA** | **1** | **(100.0) {0.1}** | **0** | **(0.0) {0.0}** | **1** | **(100.0) {0.1}** |
| **SPONDYLARTHRITE** | **1** | **(50.0) {0.1}** | **1** | **(50.0) {0.2}** | **2** | **(100.0) {0.1}** |
| **PICA/SYNDROME** | **5** | **(100.0) {0.4}** | **0** | **(0.0) {0.0}** | **5** | **(100.0) {0.3}** |
| **THYROIDECTOMIE** | **4** | **(40.0) {0.3}** | **6** | **(60.0) {1.0}** | **10** | **(100.0) {0.6}** |
| **INF/URINAIRES** | **4** | **(80.0) {0.3}** | **1** | **(20.0) {0.2}** | **5** | **(100.0) {0.3}** |
| **CATARACTE/CONG** | **2** | **(66.7) {0.2}** | **1** | **(33.3) {0.2}** | **3** | **(100.0) {0.2}** |
| **HYPERCHOLESTEROL** | **3** | **(60.0) {0.3}** | **2** | **(40.0) {0.3}** | **5** | **(100.0) {0.3}** |
| **VITILIGO** | **0** | **(0.0) {0.0}** | **1** | **(100.0) {0.2}** | **1** | **(100.0) {0.1}** |
| **OP/VES/BILIAIRE** | **4** | **(66.7) {0.3}** | **2** | **(33.3) {0.3}** | **6** | **(100.0) {0.3}** |
| **HEPATITE** | **2** | **(50.0) {0.2}** | **2** | **(50.0) {0.3}** | **4** | **(100.0) {0.2}** |
| **PHLEBITE** | **3** | **(100.0) {0.3}** | **0** | **(0.0) {0.0}** | **3** | **(100.0) {0.2}** |
| **CROHN/RCH** | **3** | **(75.0) {0.3}** | **1** | **(25.0) {0.2}** | **4** | **(100.0) {0.2}** |
| **LITHIASE/URINAIRE** | **4** | **(50.0) {0.3}** | **4** | **(50.0) {0.7}** | **8** | **(100.0) {0.5}** |
| **AVC** | **2** | **(100.0) {0.2}** | **0** | **(0.0) {0.0}** | **2** | **(100.0) {0.1}** |
| **EX/ALCOLIQUE** | **1** | **(100.0) {0.1}** | **0** | **(0.0) {0.0}** | **1** | **(100.0) {0.1}** |
| **COLIQ/NEPHRETIQ** | **3** | **(75.0) {0.3}** | **1** | **(25.0) {0.2}** | **4** | **(100.0) {0.2}** |
| **THYROIDITE** | **2** | **(66.7) {0.2}** | **1** | **(33.3) {0.2}** | **3** | **(100.0) {0.2}** |
| **DEBILITE** | **5** | **(62.5) {0.4}** | **3** | **(37.5) {0.5}** | **8** | **(100.0) {0.5}** |
| **MERE/GREFFEE** | **1** | **(100.0) {0.1}** | **0** | **(0.0) {0.0}** | **1** | **(100.0) {0.1}** |
| **HERNIE/DISCALE** | **1** | **(100.0) {0.1}** | **0** | **(0.0) {0.0}** | **1** | **(100.0) {0.1}** |
| **SYND/MALFORMATIF** | **3** | **(75.0) {0.3}** | **1** | **(25.0) {0.2}** | **4** | **(100.0) {0.2}** |
| **ANEMIE** | **5** | **(83.3) {0.4}** | **1** | **(16.7) {0.2}** | **6** | **(100.0) {0.3}** |
| **PB/VASC/CEREBRAL** | **1** | **(33.3) {0.1}** | **2** | **(66.7) {0.3}** | **3** | **(100.0) {0.2}** |
| **GASTROPLASTIE/OBESE** | **0** | **(0.0) {0.0}** | **1** | **(100.0) {0.2}** | **1** | **(100.0) {0.1}** |
| **ZAMAL** | **0** | **(0.0) {0.0}** | **2** | **(100.0) {0.3}** | **2** | **(100.0) {0.1}** |
| **OPEREE/NEUROCHIR** | **3** | **(100.0) {0.3}** | **0** | **(0.0) {0.0}** | **3** | **(100.0) {0.2}** |
| **HANDICAP** | **1** | **(100.0) {0.1}** | **0** | **(0.0) {0.0}** | **1** | **(100.0) {0.1}** |
| **DREPANOCYTOSE** | **3** | **(100.0) {0.3}** | **0** | **(0.0) {0.0}** | **3** | **(100.0) {0.2}** |
| **PANCREATITE** | **2** | **(100.0) {0.2}** | **0** | **(0.0) {0.0}** | **2** | **(100.0) {0.1}** |
| **TUBERCULOSE** | **1** | **(100.0) {0.1}** | **0** | **(0.0) {0.0}** | **1** | **(100.0) {0.1}** |
| **ANNEAU/GASTRIQUE** | **1** | **(50.0) {0.1}** | **1** | **(50.0) {0.2}** | **2** | **(100.0) {0.1}** |
| **PARABOMBAY** | **1** | **(100.0) {0.1}** | **0** | **(0.0) {0.0}** | **1** | **(100.0) {0.1}** |
| **BARIATRIQUE** | **0** | **(0.0) {0.0}** | **1** | **(100.0) {0.2}** | **1** | **(100.0) {0.1}** |
| **AUTRE** | **23** | **(74.2) {2.0}** | **8** | **(25.8) {1.4}** | **31** | **(100.0) {1.8}** |
| **Total** | **1160** | **(67.0) {100.0}** | **572** | **(33.0) {100.0}** | **1732** |  |
| **Percents: (Row) {Col}     Chi^2^= 74.290 df(60) p= 0.1015    Cells_expected<5:_ 98 (80 pct.)** | | | | | | |

**. means bmi risk /t**

**Select: (mult = 0)**

**Syntax: Means bmi /BY= risk**

| **bmi** | | | | | | | | | |
| --- | --- | --- | --- | --- | --- | --- | --- | --- | --- |
| **risk** | **Obs.** | **Sum** | **Mean** | **Variance** | **Std Dev** | **( 95% CI** | **mean )** | **Std Err** |  |
| **0** | **1096** | **29695.9** | **27.09** | **49.80** | **7.06** | **26.68** | **27.51** | **0.21** |  |
| **1** | **491** | **12972.4** | **26.42** | **35.74** | **5.98** | **25.89** | **26.95** | **0.27** |  |
|  |  |  |  |  |  |  |  |  |  |
| **risk** | **Minimum** | **p5** | **p10** | **p25** | **Median** | **p75** | **p90** | **p95** | **Max** |
| **0** | **14.50** | **18.00** | **19.50** | **21.80** | **25.60** | **31.20** | **37.23** | **40.13** | **61.10** |
| **1** | **13.10** | **17.70** | **19.52** | **22.00** | **25.70** | **30.40** | **34.70** | **37.00** | **47.10** |

| **Source** | **SS** | **df** | **MS** | **F** | **p Value** |
| --- | --- | --- | --- | --- | --- |
| **Between** | **154.24** | **1** | **154.24** | **3.39** | **0.0656** |
| **Within** | **72038.42** | **1585** | **45.45** |  |  |
| **Total** | **72192.66** | **1586** | **45.52** |  |  |
| **Bartlett's test for homogeneity of variance  Chi^2^= 17.786 df(1) p= 0.000** | | | | | |

**. tables risk bmi5 /t /o /c /r**

**Select: (mult = 0)**

| **Outcome:risk** | | | | | | |
| --- | --- | --- | --- | --- | --- | --- |
| **bmi5** | **0** | **%** | **1** | **%** | **Total** | **%** |
| **10 - 14** | **2** | **(50.0) {0.2}** | **2** | **(50.0) {0.4}** | **4** | **(100.0) {0.3}** |
| **15 - 19** | **139** | **(69.2) {12.7}** | **62** | **(30.8) {12.6}** | **201** | **(100.0) {12.7}** |
| **20 - 24** | **368** | **(69.3) {33.6}** | **163** | **(30.7) {33.2}** | **531** | **(100.0) {33.5}** |
| **25 - 29** | **256** | **(65.8) {23.4}** | **133** | **(34.2) {27.1}** | **389** | **(100.0) {24.5}** |
| **30 - 34** | **170** | **(66.7) {15.5}** | **85** | **(33.3) {17.3}** | **255** | **(100.0) {16.1}** |
| **35 - 39** | **103** | **(74.1) {9.4}** | **36** | **(25.9) {7.3}** | **139** | **(100.0) {8.8}** |
| **40 - 44** | **36** | **(83.7) {3.3}** | **7** | **(16.3) {1.4}** | **43** | **(100.0) {2.7}** |
| **45 - 49** | **16** | **(84.2) {1.5}** | **3** | **(15.8) {0.6}** | **19** | **(100.0) {1.2}** |
| **50 - 54** | **4** | **(100.0) {0.4}** | **0** | **(0.0) {0.0}** | **4** | **(100.0) {0.3}** |
| **55 - 59** | **1** | **(100.0) {0.1}** | **0** | **(0.0) {0.0}** | **1** | **(100.0) {0.1}** |
| **60 - 64** | **1** | **(100.0) {0.1}** | **0** | **(0.0) {0.0}** | **1** | **(100.0) {0.1}** |
| **Total** | **1096** | **(69.1) {100.0}** | **491** | **(30.9) {100.0}** | **1587** |  |
| **Percents: (Row) {Col}     Chi^2^= 14.010 df(10) p= 0.1725    Cells_expected<5:_ 8 (36 pct.)** | | | | | | |

**. tables risk corp /t /o /c /r**

**Select: (mult = 0)**

| **Outcome:risk** | | | | | | |
| --- | --- | --- | --- | --- | --- | --- |
| **corp** | **0** | **%** | **1** | **%** | **Total** | **%** |
| **1** | **67** | **(67.0) {6.1}** | **33** | **(33.0) {6.7}** | **100** | **(100.0) {6.3}** |
| **2** | **442** | **(69.5) {40.3}** | **194** | **(30.5) {39.5}** | **636** | **(100.0) {40.1}** |
| **3** | **256** | **(65.8) {23.4}** | **133** | **(34.2) {27.1}** | **389** | **(100.0) {24.5}** |
| **4** | **273** | **(69.3) {24.9}** | **121** | **(30.7) {24.6}** | **394** | **(100.0) {24.8}** |
| **5** | **58** | **(85.3) {5.3}** | **10** | **(14.7) {2.0}** | **68** | **(100.0) {4.3}** |
| **Total** | **1096** | **(69.1) {100.0}** | **491** | **(30.9) {100.0}** | **1587** |  |
| **Percents: (Row) {Col}     Chi^2^= 10.576 df(4) p= 0.0318** | | | | | | |

**. tables risk obes /t /o /c /r**

**Select: (mult = 0)**

| **Outcome:risk** | | | | | | |
| --- | --- | --- | --- | --- | --- | --- |
| **obes** | **1** | **%** | **0** | **%** | **Total** | **%** |
| **1** | **264** | **(31.0) {53.8}** | **587** | **(69.0) {53.6}** | **851** | **(100.0) {53.6}** |
| **0** | **227** | **(30.8) {46.2}** | **509** | **(69.2) {46.4}** | **736** | **(100.0) {46.4}** |
| **Total** | **491** | **(30.9) {100.0}** | **1096** | **(69.1) {100.0}** | **1587** |  |
| **Percents: (Row) {Col}  Exposure: obes = 1 Outcome: risk = 1    Chi^2^= 0.006 df(1) p= 0.9384    Odds Ratio = 1.01 (95% CI: 0.81-1.25)    (Robins,Greenland,Breslow CI)** | | | | | | |

**. tables risk obesplus /t /o /c /r**

**Select: (mult = 0)**

| **Outcome:risk** | | | | | | |
| --- | --- | --- | --- | --- | --- | --- |
| **obesplus** | **1** | **%** | **0** | **%** | **Total** | **%** |
| **1** | **131** | **(28.4) {26.7}** | **331** | **(71.6) {30.2}** | **462** | **(100.0) {29.1}** |
| **0** | **360** | **(32.0) {73.3}** | **765** | **(68.0) {69.8}** | **1125** | **(100.0) {70.9}** |
| **Total** | **491** | **(30.9) {100.0}** | **1096** | **(69.1) {100.0}** | **1587** |  |
| **Percents: (Row) {Col}  Exposure: obesplus = 1 Outcome: risk = 1    Chi^2^= 2.036 df(1) p= 0.1536    Odds Ratio = 0.84 (95% CI: 0.66-1.07)    (Robins,Greenland,Breslow CI)** | | | | | | |

**. tables risk thin  /t /o /c /r**

**Select: (mult = 0)**

| **Outcome:risk** | | | | | | |
| --- | --- | --- | --- | --- | --- | --- |
| **thin** | **1** | **%** | **0** | **%** | **Total** | **%** |
| **1** | **64** | **(31.2) {13.0}** | **141** | **(68.8) {12.9}** | **205** | **(100.0) {12.9}** |
| **0** | **427** | **(30.9) {87.0}** | **955** | **(69.1) {87.1}** | **1382** | **(100.0) {87.1}** |
| **Total** | **491** | **(30.9) {100.0}** | **1096** | **(69.1) {100.0}** | **1587** |  |
| **Percents: (Row) {Col}  Exposure: thin = 1 Outcome: risk = 1    Chi^2^= 0.009 df(1) p= 0.9258    Odds Ratio = 1.02 (95% CI: 0.74-1.39)    (Robins,Greenland,Breslow CI)** | | | | | | |

**. select gest > 1**

**((gest > 1) and (mult = 0))**

**. tables risk ivg /t /o /c /r**

**Select: ((gest > 1) and (mult = 0))**

| **Outcome:risk** | | | | | | |
| --- | --- | --- | --- | --- | --- | --- |
| **IVG** | **0** | **%** | **1** | **%** | **Total** | **%** |
| **0** | **549** | **(65.8) {76.6}** | **285** | **(34.2) {72.9}** | **834** | **(100.0) {75.3}** |
| **1** | **121** | **(59.0) {16.9}** | **84** | **(41.0) {21.5}** | **205** | **(100.0) {18.5}** |
| **2** | **36** | **(67.9) {5.0}** | **17** | **(32.1) {4.3}** | **53** | **(100.0) {4.8}** |
| **3** | **8** | **(66.7) {1.1}** | **4** | **(33.3) {1.0}** | **12** | **(100.0) {1.1}** |
| **4** | **3** | **(75.0) {0.4}** | **1** | **(25.0) {0.3}** | **4** | **(100.0) {0.4}** |
| **Total** | **717** | **(64.7) {100.0}** | **391** | **(35.3) {100.0}** | **1108** |  |
| **Percents: (Row) {Col}     Chi^2^= 3.803 df(4) p= 0.4333    Cells_expected<5:_ 3 (30 pct.)** | | | | | | |

**. tables risk ivgt /t /o /c /r**

**Select: ((gest > 1) and (mult = 0))**

| **Outcome:risk** | | | | | | |
| --- | --- | --- | --- | --- | --- | --- |
| **ivgt** | **1** | **%** | **0** | **%** | **Total** | **%** |
| **1** | **106** | **(38.7) {26.9}** | **168** | **(61.3) {23.0}** | **274** | **(100.0) {24.3}** |
| **0** | **288** | **(33.8) {73.1}** | **564** | **(66.2) {77.0}** | **852** | **(100.0) {75.7}** |
| **Total** | **394** | **(35.0) {100.0}** | **732** | **(65.0) {100.0}** | **1126** |  |
| **Percents: (Row) {Col}  Exposure: ivgt = 1 Outcome: risk = 1    Chi^2^= 2.173 df(1) p= 0.1404    Odds Ratio = 1.24 (95% CI: 0.93-1.64)    (Robins,Greenland,Breslow CI)** | | | | | | |

**. tables risk fcs  /t /o /c /r**

**Select: ((gest > 1) and (mult = 0))**

| **Outcome:risk** | | | | | | |
| --- | --- | --- | --- | --- | --- | --- |
| **FCS** | **0** | **%** | **1** | **%** | **Total** | **%** |
| **0** | **488** | **(64.3) {68.3}** | **271** | **(35.7) {69.3}** | **759** | **(100.0) {68.6}** |
| **1** | **161** | **(65.7) {22.5}** | **84** | **(34.3) {21.5}** | **245** | **(100.0) {22.2}** |
| **2** | **41** | **(68.3) {5.7}** | **19** | **(31.7) {4.9}** | **60** | **(100.0) {5.4}** |
| **3** | **14** | **(56.0) {2.0}** | **11** | **(44.0) {2.8}** | **25** | **(100.0) {2.3}** |
| **4** | **8** | **(61.5) {1.1}** | **5** | **(38.5) {1.3}** | **13** | **(100.0) {1.2}** |
| **5** | **1** | **(100.0) {0.1}** | **0** | **(0.0) {0.0}** | **1** | **(100.0) {0.1}** |
| **6** | **1** | **(50.0) {0.1}** | **1** | **(50.0) {0.3}** | **2** | **(100.0) {0.2}** |
| **9** | **1** | **(100.0) {0.1}** | **0** | **(0.0) {0.0}** | **1** | **(100.0) {0.1}** |
| **Total** | **715** | **(64.6) {100.0}** | **391** | **(35.4) {100.0}** | **1106** |  |
| **Percents: (Row) {Col}     Chi^2^= 2.674 df(7) p= 0.9134    Cells_expected<5:_ 7 (44 pct.)** | | | | | | |

**. tables risk fcst /t /o /c /r**

**Select: ((gest > 1) and (mult = 0))**

| **Outcome:risk** | | | | | | |
| --- | --- | --- | --- | --- | --- | --- |
| **fcst** | **1** | **%** | **0** | **%** | **Total** | **%** |
| **1** | **120** | **(34.6) {30.5}** | **227** | **(65.4) {31.0}** | **347** | **(100.0) {30.8}** |
| **0** | **274** | **(35.2) {69.5}** | **505** | **(64.8) {69.0}** | **779** | **(100.0) {69.2}** |
| **Total** | **394** | **(35.0) {100.0}** | **732** | **(65.0) {100.0}** | **1126** |  |
| **Percents: (Row) {Col}  Exposure: fcst = 1 Outcome: risk = 1    Chi^2^= 0.037 df(1) p= 0.8477    Odds Ratio = 0.97 (95% CI: 0.75-1.27)    (Robins,Greenland,Breslow CI)** | | | | | | |

**. tables risk atces /t /o /c /r**

**Select: ((gest > 1) and (mult = 0))**

| **Outcome:risk** | | | | | | |
| --- | --- | --- | --- | --- | --- | --- |
| **atces** | **1** | **%** | **0** | **%** | **Total** | **%** |
| **1** | **101** | **(37.5) {32.2}** | **168** | **(62.5) {28.7}** | **269** | **(100.0) {29.9}** |
| **0** | **213** | **(33.8) {67.8}** | **417** | **(66.2) {71.3}** | **630** | **(100.0) {70.1}** |
| **Total** | **314** | **(34.9) {100.0}** | **585** | **(65.1) {100.0}** | **899** |  |
| **Percents: (Row) {Col}  Exposure: atces = 1 Outcome: risk = 1    Chi^2^= 1.158 df(1) p= 0.2818    Odds Ratio = 1.18 (95% CI: 0.87-1.58)    (Robins,Greenland,Breslow CI)** | | | | | | |

**. tables risk dcperi /t /o /c /r**

**Select: ((gest > 1) and (mult = 0))**

| **Outcome:risk** | | | | | | |
| --- | --- | --- | --- | --- | --- | --- |
| **dcperinatals** | **0** | **%** | **1** | **%** | **Total** | **%** |
| **0** | **661** | **(65.9) {92.6}** | **342** | **(34.1) {87.5}** | **1003** | **(100.0) {90.8}** |
| **1** | **47** | **(51.1) {6.6}** | **45** | **(48.9) {11.5}** | **92** | **(100.0) {8.3}** |
| **2** | **4** | **(57.1) {0.6}** | **3** | **(42.9) {0.8}** | **7** | **(100.0) {0.6}** |
| **3** | **2** | **(66.7) {0.3}** | **1** | **(33.3) {0.3}** | **3** | **(100.0) {0.3}** |
| **Total** | **714** | **(64.6) {100.0}** | **391** | **(35.4) {100.0}** | **1105** |  |
| **Percents: (Row) {Col}     Chi^2^= 8.267 df(3) p= 0.0408    Cells_expected<5:_ 4 (50 pct.)** | | | | | | |

**. tables risk cesar /t /o /c /r**

**Select: ((gest > 1) and (mult = 0))**

| **Outcome:risk** | | | | | | |
| --- | --- | --- | --- | --- | --- | --- |
| **Atcd Cesar** | **0** | **%** | **1** | **%** | **Total** | **%** |
| **0** | **522** | **(65.7) {76.0}** | **273** | **(34.3) {73.0}** | **795** | **(100.0) {74.9}** |
| **1** | **127** | **(66.8) {18.5}** | **63** | **(33.2) {16.8}** | **190** | **(100.0) {17.9}** |
| **2** | **25** | **(50.0) {3.6}** | **25** | **(50.0) {6.7}** | **50** | **(100.0) {4.7}** |
| **3** | **11** | **(55.0) {1.6}** | **9** | **(45.0) {2.4}** | **20** | **(100.0) {1.9}** |
| **4** | **2** | **(40.0) {0.3}** | **3** | **(60.0) {0.8}** | **5** | **(100.0) {0.5}** |
| **5** | **0** | **(0.0) {0.0}** | **1** | **(100.0) {0.3}** | **1** | **(100.0) {0.1}** |
| **Total** | **687** | **(64.8) {100.0}** | **374** | **(35.2) {100.0}** | **1061** |  |
| **Percents: (Row) {Col}     Chi^2^= 9.431 df(5) p= 0.0931    Cells_expected<5:_ 4 (33 pct.)** | | | | | | |

**. select**

**. select mult = 0**

**. means prise risk /t**

**Select: (mult = 0)**

**Syntax: Means PRISE /BY= risk**

| **kg Prise poids** | | | | | | | | | |
| --- | --- | --- | --- | --- | --- | --- | --- | --- | --- |
| **risk** | **Obs.** | **Sum** | **Mean** | **Variance** | **Std Dev** | **( 95% CI** | **mean )** | **Std Err** |  |
| **0** | **982** | **13422.0** | **13.67** | **51.91** | **7.20** | **13.22** | **14.12** | **0.23** |  |
| **1** | **378** | **3998.00** | **10.58** | **41.09** | **6.41** | **9.93** | **11.22** | **0.33** |  |
|  |  |  |  |  |  |  |  |  |  |
| **risk** | **Minimum** | **p5** | **p10** | **p25** | **Median** | **p75** | **p90** | **p95** | **Max** |
| **0** | **-8.00** | **2.15** | **5.00** | **9.00** | **13.00** | **18.00** | **23.00** | **26.00** | **48.00** |
| **1** | **-7.00** | **2.00** | **3.00** | **6.00** | **10.00** | **14.00** | **18.10** | **21.00** | **45.00** |

| **Source** | **SS** | **df** | **MS** | **F** | **p Value** |
| --- | --- | --- | --- | --- | --- |
| **Between** | **2608.24** | **1** | **2608.24** | **53.33** | **0.00000000** |
| **Within** | **66412.05** | **1358** | **48.90** |  |  |
| **Total** | **69020.29** | **1359** | **50.79** |  |  |
| **Bartlett's test for homogeneity of variance  Chi^2^= 7.168 df(1) p= 0.007** | | | | | |

**. means cs risk /t**

**Select: (mult = 0)**

**Syntax: Means CS /BY= risk**

| **Nb consultations** | | | | | | | | | |
| --- | --- | --- | --- | --- | --- | --- | --- | --- | --- |
| **risk** | **Obs.** | **Sum** | **Mean** | **Variance** | **Std Dev** | **( 95% CI** | **mean )** | **Std Err** |  |
| **0** | **1122** | **9910.0** | **8.83** | **7.56** | **2.75** | **8.67** | **8.99** | **0.08** |  |
| **1** | **538** | **3494.00** | **6.49** | **6.72** | **2.59** | **6.27** | **6.71** | **0.11** |  |
|  |  |  |  |  |  |  |  |  |  |
| **risk** | **Minimum** | **p5** | **p10** | **p25** | **Median** | **p75** | **p90** | **p95** | **Max** |
| **0** | **0.0** | **5.00** | **6.00** | **7.00** | **8.00** | **10.00** | **12.00** | **14.00** | **32.00** |
| **1** | **0.0** | **2.00** | **4.00** | **5.00** | **6.00** | **8.00** | **10.00** | **11.00** | **24.00** |

| **Source** | **SS** | **df** | **MS** | **F** | **p Value** |
| --- | --- | --- | --- | --- | --- |
| **Between** | **1987.75** | **1** | **1987.75** | **272.85** | **0.00000000** |
| **Within** | **12078.98** | **1658** | **7.29** |  |  |
| **Total** | **14066.74** | **1659** | **8.48** |  |  |
| **Bartlett's test for homogeneity of variance  Chi^2^= 2.494 df(1) p= 0.114** | | | | | |

**. tables risk c3 /t /o /c /r**

**Select: (mult = 0)**

| **Outcome:risk** | | | | | | |
| --- | --- | --- | --- | --- | --- | --- |
| **c3** | **1** | **%** | **0** | **%** | **Total** | **%** |
| **1** | **49** | **(84.5) {9.1}** | **9** | **(15.5) {0.8}** | **58** | **(100.0) {3.5}** |
| **0** | **489** | **(30.5) {90.9}** | **1113** | **(69.5) {99.2}** | **1602** | **(100.0) {96.5}** |
| **Total** | **538** | **(32.4) {100.0}** | **1122** | **(67.6) {100.0}** | **1660** |  |
| **Percents: (Row) {Col}  Exposure: c3 = 1 Outcome: risk = 1    Chi^2^= 74.395 df(1) p= 0.0000    Odds Ratio = 12.39 (95% CI: 6.04-25.43)    (Robins,Greenland,Breslow CI)** | | | | | | |

**. means TERMNOT risk /t**

**Select: (mult = 0)**

**Syntax: Means TERMNOT /BY= risk**

| **Termenot‚** | | | | | | | | | |
| --- | --- | --- | --- | --- | --- | --- | --- | --- | --- |
| **risk** | **Obs.** | **Sum** | **Mean** | **Variance** | **Std Dev** | **( 95% CI** | **mean )** | **Std Err** |  |
| **0** | **1109** | **12491.0** | **11.26** | **14.81** | **3.85** | **11.04** | **11.49** | **0.12** |  |
| **1** | **525** | **5997.0** | **11.42** | **17.31** | **4.16** | **11.07** | **11.78** | **0.18** |  |
|  |  |  |  |  |  |  |  |  |  |
| **risk** | **Minimum** | **p5** | **p10** | **p25** | **Median** | **p75** | **p90** | **p95** | **Max** |
| **0** | **0.0** | **6.00** | **7.00** | **9.00** | **12.00** | **13.00** | **14.00** | **18.00** | **36.00** |
| **1** | **5.00** | **6.00** | **7.00** | **9.00** | **12.00** | **13.00** | **16.00** | **20.70** | **30.00** |

| **Source** | **SS** | **df** | **MS** | **F** | **p Value** |
| --- | --- | --- | --- | --- | --- |
| **Between** | **9.07** | **1** | **9.07** | **0.58** | **0.446** |
| **Within** | **25479.24** | **1632** | **15.61** |  |  |
| **Total** | **25488.31** | **1633** | **15.61** |  |  |
| **Bartlett's test for homogeneity of variance  Chi^2^= 4.381 df(1) p= 0.036** | | | | | |

**. tables risk rub /t /o /c /r**

**Select: (mult = 0)**

| **Outcome:risk** | | | | | | |
| --- | --- | --- | --- | --- | --- | --- |
| **Rub‚ole** | **0** | **%** | **1** | **%** | **Total** | **%** |
| **0** | **72** | **(68.6) {6.2}** | **33** | **(31.4) {5.7}** | **105** | **(100.0) {6.0}** |
| **1** | **1083** | **(67.2) {93.2}** | **528** | **(32.8) {92.0}** | **1611** | **(100.0) {92.8}** |
| **9** | **7** | **(35.0) {0.6}** | **13** | **(65.0) {2.3}** | **20** | **(100.0) {1.2}** |
| **Total** | **1162** | **(66.9) {100.0}** | **574** | **(33.1) {100.0}** | **1736** |  |
| **Percents: (Row) {Col}     Chi^2^= 9.404 df(2) p= 0.0091** | | | | | | |

**. tables risk toxo /t /o /c /r**

**Select: (mult = 0)**

| **Outcome:risk** | | | | | | |
| --- | --- | --- | --- | --- | --- | --- |
| **Toxoplasmose** | **0** | **%** | **1** | **%** | **Total** | **%** |
| **0** | **584** | **(67.0) {50.3}** | **287** | **(33.0) {50.0}** | **871** | **(100.0) {50.2}** |
| **1** | **572** | **(67.5) {49.2}** | **276** | **(32.5) {48.1}** | **848** | **(100.0) {48.8}** |
| **9** | **6** | **(35.3) {0.5}** | **11** | **(64.7) {1.9}** | **17** | **(100.0) {1.0}** |
| **Total** | **1162** | **(66.9) {100.0}** | **574** | **(33.1) {100.0}** | **1736** |  |
| **Percents: (Row) {Col}     Chi^2^= 7.798 df(2) p= 0.0203** | | | | | | |

**. tables risk bw /t /o /c /r**

**Select: (mult = 0)**

| **Outcome:risk** | | | | | | |
| --- | --- | --- | --- | --- | --- | --- |
| **BW** | **0** | **%** | **1** | **%** | **Total** | **%** |
| **0** | **1145** | **(67.7) {98.5}** | **547** | **(32.3) {95.3}** | **1692** | **(100.0) {97.5}** |
| **1** | **5** | **(62.5) {0.4}** | **3** | **(37.5) {0.5}** | **8** | **(100.0) {0.5}** |
| **9** | **12** | **(33.3) {1.0}** | **24** | **(66.7) {4.2}** | **36** | **(100.0) {2.1}** |
| **Total** | **1162** | **(66.9) {100.0}** | **574** | **(33.1) {100.0}** | **1736** |  |
| **Percents: (Row) {Col}     Chi^2^= 18.851 df(2) p= 0.0001    Cells_expected<5:_ 1 (17 pct.)** | | | | | | |

**. tables risk hbs /t /o /c /r**

**Select: (mult = 0)**

| **Outcome:risk** | | | | | | |
| --- | --- | --- | --- | --- | --- | --- |
| **HBS** | **0** | **%** | **1** | **%** | **Total** | **%** |
| **0** | **1132** | **(68.4) {97.4}** | **522** | **(31.6) {90.9}** | **1654** | **(100.0) {95.3}** |
| **1** | **8** | **(57.1) {0.7}** | **6** | **(42.9) {1.0}** | **14** | **(100.0) {0.8}** |
| **9** | **22** | **(32.4) {1.9}** | **46** | **(67.6) {8.0}** | **68** | **(100.0) {3.9}** |
| **Total** | **1162** | **(66.9) {100.0}** | **574** | **(33.1) {100.0}** | **1736** |  |
| **Percents: (Row) {Col}     Chi^2^= 39.044 df(2) p= 0.0000    Cells_expected<5:_ 1 (17 pct.)** | | | | | | |

**. tables risk hiv /t /o /c /r**

**Select: (mult = 0)**

| **Outcome:risk** | | | | | | |
| --- | --- | --- | --- | --- | --- | --- |
| **HIV** | **1** | **%** | **0** | **%** | **Total** | **%** |
| **9** | **28** | **(59.6) {4.9}** | **19** | **(40.4) {1.6}** | **47** | **(100.0) {2.7}** |
| **0** | **546** | **(32.3) {95.1}** | **1143** | **(67.7) {98.4}** | **1689** | **(100.0) {97.3}** |
| **Total** | **574** | **(33.1) {100.0}** | **1162** | **(66.9) {100.0}** | **1736** |  |
| **Percents: (Row) {Col}  Exposure: HIV = 9 Outcome: risk = 1    Chi^2^= 15.340 df(1) p= 0.0001    Odds Ratio = 3.09 (95% CI: 1.71-5.57)    (Robins,Greenland,Breslow CI)** | | | | | | |

**. tables risk rai /t /o /c /r**

**Select: (mult = 0)**

| **Outcome:risk** | | | | | | |
| --- | --- | --- | --- | --- | --- | --- |
| **RAI** | **0** | **%** | **1** | **%** | **Total** | **%** |
| **NEGATIF** | **860** | **(71.8) {86.3}** | **338** | **(28.2) {74.1}** | **1198** | **(100.0) {82.5}** |
| **POSITIF** | **10** | **(71.4) {1.0}** | **4** | **(28.6) {0.9}** | **14** | **(100.0) {1.0}** |
| **NON/FAIT** | **127** | **(52.7) {12.7}** | **114** | **(47.3) {25.0}** | **241** | **(100.0) {16.6}** |
| **Total** | **997** | **(68.6) {100.0}** | **456** | **(31.4) {100.0}** | **1453** |  |
| **Percents: (Row) {Col}     Chi^2^= 34.004 df(2) p= 0.0000    Cells_expected<5:_ 1 (17 pct.)** | | | | | | |

**. tables risk hgie /t /o /c /r**

**Select: (mult = 0)**

| **Outcome:risk** | | | | | | |
| --- | --- | --- | --- | --- | --- | --- |
| **H‚morragie d‚livrance** | **0** | **%** | **1** | **%** | **Total** | **%** |
| **NON** | **944** | **(68.0) {94.7}** | **445** | **(32.0) {97.8}** | **1389** | **(100.0) {95.7}** |
| **SYNTO/RU** | **25** | **(86.2) {2.5}** | **4** | **(13.8) {0.9}** | **29** | **(100.0) {2.0}** |
| **PROSTAGL** | **19** | **(82.6) {1.9}** | **4** | **(17.4) {0.9}** | **23** | **(100.0) {1.6}** |
| **EMBOL** | **7** | **(77.8) {0.7}** | **2** | **(22.2) {0.4}** | **9** | **(100.0) {0.6}** |
| **LIG/CHIR** | **1** | **(100.0) {0.1}** | **0** | **(0.0) {0.0}** | **1** | **(100.0) {0.1}** |
| **HYSTERECT** | **1** | **(100.0) {0.1}** | **0** | **(0.0) {0.0}** | **1** | **(100.0) {0.1}** |
| **Total** | **997** | **(68.7) {100.0}** | **455** | **(31.3) {100.0}** | **1452** |  |
| **Percents: (Row) {Col}     Chi^2^= 7.804 df(5) p= 0.1674    Cells_expected<5:_ 5 (42 pct.)** | | | | | | |

**. tables risk ru /t /o /c /r**

**Select: (mult = 0)**

| **Outcome:risk** | | | | | | |
| --- | --- | --- | --- | --- | --- | --- |
| **R‚vision Ut‚rine** | **1** | **%** | **0** | **%** | **Total** | **%** |
| **OUI** | **64** | **(34.0) {14.1}** | **124** | **(66.0) {12.4}** | **188** | **(100.0) {12.9}** |
| **NON** | **391** | **(30.9) {85.9}** | **874** | **(69.1) {87.6}** | **1265** | **(100.0) {87.1}** |
| **Total** | **455** | **(31.3) {100.0}** | **998** | **(68.7) {100.0}** | **1453** |  |
| **Percents: (Row) {Col}  Exposure: R‚vision Ut‚rine = OUI Outcome: risk = 1    Chi^2^= 0.747 df(1) p= 0.3874    Odds Ratio = 1.15 (95% CI: 0.83-1.60)    (Robins,Greenland,Breslow CI)** | | | | | | |

**. tables risk epis /t /o /c /r**

**Select: (mult = 0)**

| **Outcome:risk** | | | | | | |
| --- | --- | --- | --- | --- | --- | --- |
| **Episiotomie** | **1** | **%** | **0** | **%** | **Total** | **%** |
| **OUI** | **2** | **(1.6) {0.4}** | **125** | **(98.4) {12.5}** | **127** | **(100.0) {8.7}** |
| **NON** | **453** | **(34.2) {99.6}** | **873** | **(65.8) {87.5}** | **1326** | **(100.0) {91.3}** |
| **Total** | **455** | **(31.3) {100.0}** | **998** | **(68.7) {100.0}** | **1453** |  |
| **Percents: (Row) {Col}  Exposure: Episiotomie = OUI Outcome: risk = 1    Chi^2^= 57.225 df(1) p= 0.0000    Odds Ratio = 0.03 (95% CI: 0.01-0.13)    (Robins,Greenland,Breslow CI)** | | | | | | |

**. tables risk gyn /t /o /c /r**

**Select: (mult = 0)**

| **Outcome:risk** | | | | | | |
| --- | --- | --- | --- | --- | --- | --- |
| **Pathologie gyn‚co** | **0** | **%** | **1** | **%** | **Total** | **%** |
| **0** | **884** | **(69.5) {76.2}** | **388** | **(30.5) {68.0}** | **1272** | **(100.0) {73.5}** |
| **1** | **11** | **(61.1) {0.9}** | **7** | **(38.9) {1.2}** | **18** | **(100.0) {1.0}** |
| **2** | **2** | **(50.0) {0.2}** | **2** | **(50.0) {0.4}** | **4** | **(100.0) {0.2}** |
| **3** | **2** | **(33.3) {0.2}** | **4** | **(66.7) {0.7}** | **6** | **(100.0) {0.3}** |
| **4** | **17** | **(58.6) {1.5}** | **12** | **(41.4) {2.1}** | **29** | **(100.0) {1.7}** |
| **5** | **1** | **(100.0) {0.1}** | **0** | **(0.0) {0.0}** | **1** | **(100.0) {0.1}** |
| **6** | **1** | **(100.0) {0.1}** | **0** | **(0.0) {0.0}** | **1** | **(100.0) {0.1}** |
| **8** | **0** | **(0.0) {0.0}** | **1** | **(100.0) {0.2}** | **1** | **(100.0) {0.1}** |
| **9** | **5** | **(83.3) {0.4}** | **1** | **(16.7) {0.2}** | **6** | **(100.0) {0.3}** |
| **SALPINGECTOMIE** | **4** | **(66.7) {0.3}** | **2** | **(33.3) {0.4}** | **6** | **(100.0) {0.3}** |
| **ATCD/GEU** | **1** | **(100.0) {0.1}** | **0** | **(0.0) {0.0}** | **1** | **(100.0) {0.1}** |
| **ATCD/PREECLAMPSIE** | **70** | **(53.4) {6.0}** | **61** | **(46.6) {10.7}** | **131** | **(100.0) {7.6}** |
| **ATCD/MIU** | **23** | **(56.1) {2.0}** | **18** | **(43.9) {3.2}** | **41** | **(100.0) {2.4}** |
| **UT/CICATRICIEL** | **18** | **(100.0) {1.6}** | **0** | **(0.0) {0.0}** | **18** | **(100.0) {1.0}** |
| **MALF/UTERINE** | **3** | **(75.0) {0.3}** | **1** | **(25.0) {0.2}** | **4** | **(100.0) {0.2}** |
| **ATCD/ACC/PREMA** | **15** | **(57.7) {1.3}** | **11** | **(42.3) {1.9}** | **26** | **(100.0) {1.5}** |
| **ATCD/RCIU** | **5** | **(62.5) {0.4}** | **3** | **(37.5) {0.5}** | **8** | **(100.0) {0.5}** |
| **ATCD/DIAB/GEST** | **4** | **(80.0) {0.3}** | **1** | **(20.0) {0.2}** | **5** | **(100.0) {0.3}** |
| **ATCD/THROMBOPENIE** | **1** | **(100.0) {0.1}** | **0** | **(0.0) {0.0}** | **1** | **(100.0) {0.1}** |
| **MYOMECTOMIE** | **3** | **(60.0) {0.3}** | **2** | **(40.0) {0.4}** | **5** | **(100.0) {0.3}** |
| **ATCD/STREPTOB** | **16** | **(80.0) {1.4}** | **4** | **(20.0) {0.7}** | **20** | **(100.0) {1.2}** |
| **ATCD/EXTRACTION** | **12** | **(85.7) {1.0}** | **2** | **(14.3) {0.4}** | **14** | **(100.0) {0.8}** |
| **HERPES** | **10** | **(83.3) {0.9}** | **2** | **(16.7) {0.4}** | **12** | **(100.0) {0.7}** |
| **FIBROME** | **8** | **(42.1) {0.7}** | **11** | **(57.9) {1.9}** | **19** | **(100.0) {1.1}** |
| **G/STIMULEE** | **3** | **(37.5) {0.3}** | **5** | **(62.5) {0.9}** | **8** | **(100.0) {0.5}** |
| **ATCD/ITG** | **7** | **(50.0) {0.6}** | **7** | **(50.0) {1.2}** | **14** | **(100.0) {0.8}** |
| **THROMBOPENIE** | **2** | **(100.0) {0.2}** | **0** | **(0.0) {0.0}** | **2** | **(100.0) {0.1}** |
| **BARTHOLINITE** | **0** | **(0.0) {0.0}** | **1** | **(100.0) {0.2}** | **1** | **(100.0) {0.1}** |
| **PERMEABILø/TUBAIRE** | **1** | **(100.0) {0.1}** | **0** | **(0.0) {0.0}** | **1** | **(100.0) {0.1}** |
| **CONISATION/COL** | **2** | **(50.0) {0.2}** | **2** | **(50.0) {0.4}** | **4** | **(100.0) {0.2}** |
| **G/SS/STERILET** | **1** | **(100.0) {0.1}** | **0** | **(0.0) {0.0}** | **1** | **(100.0) {0.1}** |
| **ATCD/MALFORMATø** | **4** | **(50.0) {0.3}** | **4** | **(50.0) {0.7}** | **8** | **(100.0) {0.5}** |
| **ENDOMETRITE** | **0** | **(0.0) {0.0}** | **1** | **(100.0) {0.2}** | **1** | **(100.0) {0.1}** |
| **CONDYLOMES** | **4** | **(100.0) {0.3}** | **0** | **(0.0) {0.0}** | **4** | **(100.0) {0.2}** |
| **CHIRURGIE/MAMMAIRE** | **1** | **(100.0) {0.1}** | **0** | **(0.0) {0.0}** | **1** | **(100.0) {0.1}** |
| **ATCD/GMOLAIRE** | **2** | **(50.0) {0.2}** | **2** | **(50.0) {0.4}** | **4** | **(100.0) {0.2}** |
| **ATCD/PERINEE** | **1** | **(100.0) {0.1}** | **0** | **(0.0) {0.0}** | **1** | **(100.0) {0.1}** |
| **ATCD/G/MULTIPLE** | **13** | **(72.2) {1.1}** | **5** | **(27.8) {0.9}** | **18** | **(100.0) {1.0}** |
| **ATCD/OEUF/CLAIR** | **1** | **(33.3) {0.1}** | **2** | **(66.7) {0.4}** | **3** | **(100.0) {0.2}** |
| **G/SOUS/PILULE** | **0** | **(0.0) {0.0}** | **2** | **(100.0) {0.4}** | **2** | **(100.0) {0.1}** |
| **ICSI** | **0** | **(0.0) {0.0}** | **2** | **(100.0) {0.4}** | **2** | **(100.0) {0.1}** |
| **INSEMINATION** | **1** | **(100.0) {0.1}** | **0** | **(0.0) {0.0}** | **1** | **(100.0) {0.1}** |
| **61** | **0** | **(0.0) {0.0}** | **1** | **(100.0) {0.2}** | **1** | **(100.0) {0.1}** |
| **AUTRE** | **1** | **(20.0) {0.1}** | **4** | **(80.0) {0.7}** | **5** | **(100.0) {0.3}** |
| **Total** | **1160** | **(67.0) {100.0}** | **571** | **(33.0) {100.0}** | **1731** |  |
| **Percents: (Row) {Col}     Chi^2^= 80.778 df(43) p= 0.0004    Cells_expected<5:_ 62 (70 pct.)** | | | | | | |

**. tables risk diabete /t /o /c /r**

**Select: (mult = 0)**

| **Outcome:risk** | | | | | | |
| --- | --- | --- | --- | --- | --- | --- |
| **g/l Diabete** | **1** | **%** | **0** | **%** | **Total** | **%** |
| **1** | **73** | **(26.4) {12.7}** | **204** | **(73.6) {17.6}** | **277** | **(100.0) {16.0}** |
| **0** | **501** | **(34.4) {87.3}** | **957** | **(65.6) {82.4}** | **1458** | **(100.0) {84.0}** |
| **Total** | **574** | **(33.1) {100.0}** | **1161** | **(66.9) {100.0}** | **1735** |  |
| **Percents: (Row) {Col}  Exposure: g/l Diabete = 1 Outcome: risk = 1    Chi^2^= 6.743 df(1) p= 0.0094    Odds Ratio = 0.68 (95% CI: 0.51-0.91)    (Robins,Greenland,Breslow CI)** | | | | | | |

**. tables risk diabgest /t /o /c /r**

**Select: (mult = 0)**

| **Outcome:risk** | | | | | | |
| --- | --- | --- | --- | --- | --- | --- |
| **diabgest** | **1** | **%** | **0** | **%** | **Total** | **%** |
| **1** | **66** | **(26.1) {11.7}** | **187** | **(73.9) {16.4}** | **253** | **(100.0) {14.9}** |
| **0** | **496** | **(34.2) {88.3}** | **953** | **(65.8) {83.6}** | **1449** | **(100.0) {85.1}** |
| **Total** | **562** | **(33.0) {100.0}** | **1140** | **(67.0) {100.0}** | **1702** |  |
| **Percents: (Row) {Col}  Exposure: diabgest = 1 Outcome: risk = 1    Chi^2^= 6.459 df(1) p= 0.0110    Odds Ratio = 0.68 (95% CI: 0.50-0.92)    (Robins,Greenland,Breslow CI)** | | | | | | |

**. tables risk diabprex /t /o /c /r**

**Select: (mult = 0)**

| **Outcome:risk** | | | | | | |
| --- | --- | --- | --- | --- | --- | --- |
| **diabprex** | **1** | **%** | **0** | **%** | **Total** | **%** |
| **1** | **20** | **(30.3) {3.9}** | **46** | **(69.7) {4.6}** | **66** | **(100.0) {4.4}** |
| **0** | **496** | **(34.2) {96.1}** | **953** | **(65.8) {95.4}** | **1449** | **(100.0) {95.6}** |
| **Total** | **516** | **(34.1) {100.0}** | **999** | **(65.9) {100.0}** | **1515** |  |
| **Percents: (Row) {Col}  Exposure: diabprex = 1 Outcome: risk = 1    Chi^2^= 0.434 df(1) p= 0.5103    Odds Ratio = 0.84 (95% CI: 0.49-1.43)    (Robins,Greenland,Breslow CI)** | | | | | | |

**. tables risk insuline /t /o /c /r**

**Select: (mult = 0)**

| **Outcome:risk** | | | | | | |
| --- | --- | --- | --- | --- | --- | --- |
| **insuline** | **1** | **%** | **0** | **%** | **Total** | **%** |
| **1** | **44** | **(29.1) {7.7}** | **107** | **(70.9) {9.2}** | **151** | **(100.0) {8.7}** |
| **0** | **530** | **(33.4) {92.3}** | **1055** | **(66.6) {90.8}** | **1585** | **(100.0) {91.3}** |
| **Total** | **574** | **(33.1) {100.0}** | **1162** | **(66.9) {100.0}** | **1736** |  |
| **Percents: (Row) {Col}  Exposure: insuline = 1 Outcome: risk = 1    Chi^2^= 1.151 df(1) p= 0.2832    Odds Ratio = 0.82 (95% CI: 0.57-1.18)    (Robins,Greenland,Breslow CI)** | | | | | | |

**. select diabgest = 1**

**((diabgest = 1) and (mult = 0))**

**. tables risk insuline /t /o /c /r**

**Select: ((diabgest = 1) and (mult = 0))**

| **Outcome:risk** | | | | | | |
| --- | --- | --- | --- | --- | --- | --- |
| **insuline** | **1** | **%** | **0** | **%** | **Total** | **%** |
| **1** | **34** | **(27.9) {51.5}** | **88** | **(72.1) {47.1}** | **122** | **(100.0) {48.2}** |
| **0** | **32** | **(24.4) {48.5}** | **99** | **(75.6) {52.9}** | **131** | **(100.0) {51.8}** |
| **Total** | **66** | **(26.1) {100.0}** | **187** | **(73.9) {100.0}** | **253** |  |
| **Percents: (Row) {Col}  Exposure: insuline = 1 Outcome: risk = 1    Chi^2^= 0.388 df(1) p= 0.5334    Odds Ratio = 1.20 (95% CI: 0.68-2.10)    (Robins,Greenland,Breslow CI)** | | | | | | |

**. select**

**. select mult = 0**

**. tables risk hosprisk /t /o /c /r**

**Select: (mult = 0)**

| **Outcome:risk** | | | | | | |
| --- | --- | --- | --- | --- | --- | --- |
| **hosprisk** | **1** | **%** | **0** | **%** | **Total** | **%** |
| **1** | **484** | **(42.8) {84.3}** | **646** | **(57.2) {55.6}** | **1130** | **(100.0) {65.1}** |
| **0** | **90** | **(14.9) {15.7}** | **516** | **(85.1) {44.4}** | **606** | **(100.0) {34.9}** |
| **Total** | **574** | **(33.1) {100.0}** | **1162** | **(66.9) {100.0}** | **1736** |  |
| **Percents: (Row) {Col}  Exposure: hosprisk = 1 Outcome: risk = 1    Chi^2^=139.537 df(1) p= 0.0000    Odds Ratio = 4.30 (95% CI: 3.34-5.53)    (Robins,Greenland,Breslow CI)** | | | | | | |

**. tables risk motif /t /o /c /r**

**Select: (mult = 0)**

| **Outcome:risk** | | | | | | |
| --- | --- | --- | --- | --- | --- | --- |
| **motif** | **0** | **%** | **1** | **%** | **Total** | **%** |
| **1** | **9** | **(75.0) {1.4}** | **3** | **(25.0) {0.6}** | **12** | **(100.0) {1.1}** |
| **2** | **553** | **(58.3) {86.0}** | **395** | **(41.7) {81.6}** | **948** | **(100.0) {84.1}** |
| **3** | **41** | **(37.3) {6.4}** | **69** | **(62.7) {14.3}** | **110** | **(100.0) {9.8}** |
| **4** | **2** | **(100.0) {0.3}** | **0** | **(0.0) {0.0}** | **2** | **(100.0) {0.2}** |
| **5** | **1** | **(25.0) {0.2}** | **3** | **(75.0) {0.6}** | **4** | **(100.0) {0.4}** |
| **7** | **7** | **(87.5) {1.1}** | **1** | **(12.5) {0.2}** | **8** | **(100.0) {0.7}** |
| **10** | **4** | **(100.0) {0.6}** | **0** | **(0.0) {0.0}** | **4** | **(100.0) {0.4}** |
| **12** | **1** | **(16.7) {0.2}** | **5** | **(83.3) {1.0}** | **6** | **(100.0) {0.5}** |
| **13** | **1** | **(100.0) {0.2}** | **0** | **(0.0) {0.0}** | **1** | **(100.0) {0.1}** |
| **14** | **2** | **(66.7) {0.3}** | **1** | **(33.3) {0.2}** | **3** | **(100.0) {0.3}** |
| **15** | **0** | **(0.0) {0.0}** | **1** | **(100.0) {0.2}** | **1** | **(100.0) {0.1}** |
| **18** | **3** | **(60.0) {0.5}** | **2** | **(40.0) {0.4}** | **5** | **(100.0) {0.4}** |
| **19** | **1** | **(100.0) {0.2}** | **0** | **(0.0) {0.0}** | **1** | **(100.0) {0.1}** |
| **20** | **0** | **(0.0) {0.0}** | **1** | **(100.0) {0.2}** | **1** | **(100.0) {0.1}** |
| **22** | **13** | **(92.9) {2.0}** | **1** | **(7.1) {0.2}** | **14** | **(100.0) {1.2}** |
| **25** | **1** | **(100.0) {0.2}** | **0** | **(0.0) {0.0}** | **1** | **(100.0) {0.1}** |
| **28** | **1** | **(100.0) {0.2}** | **0** | **(0.0) {0.0}** | **1** | **(100.0) {0.1}** |
| **33** | **2** | **(50.0) {0.3}** | **2** | **(50.0) {0.4}** | **4** | **(100.0) {0.4}** |
| **35** | **1** | **(100.0) {0.2}** | **0** | **(0.0) {0.0}** | **1** | **(100.0) {0.1}** |
| **Total** | **643** | **(57.1) {100.0}** | **484** | **(42.9) {100.0}** | **1127** |  |
| **Percents: (Row) {Col}     Chi^2^= 46.949 df(18) p= 0.0002    Cells_expected<5:_ 30 (79 pct.)** | | | | | | |

**. tables risk hj /t /o /c /r**

**Select: (mult = 0)**

| **Outcome:risk** | | | | | | |
| --- | --- | --- | --- | --- | --- | --- |
| **hj** | **1** | **%** | **0** | **%** | **Total** | **%** |
| **1** | **37** | **(16.1) {6.8}** | **193** | **(83.9) {17.3}** | **230** | **(100.0) {13.8}** |
| **0** | **510** | **(35.6) {93.2}** | **922** | **(64.4) {82.7}** | **1432** | **(100.0) {86.2}** |
| **Total** | **547** | **(32.9) {100.0}** | **1115** | **(67.1) {100.0}** | **1662** |  |
| **Percents: (Row) {Col}  Exposure: hj = 1 Outcome: risk = 1    Chi^2^= 34.224 df(1) p= 0.0000    Odds Ratio = 0.35 (95% CI: 0.24-0.50)    (Robins,Greenland,Breslow CI)** | | | | | | |

**. tables risk mothj /t /o /c /r**

**Select: (mult = 0)**

| **Outcome:risk** | | | | | | |
| --- | --- | --- | --- | --- | --- | --- |
| **mothj** | **0** | **%** | **1** | **%** | **Total** | **%** |
| **1** | **1** | **(100.0) {0.5}** | **0** | **(0.0) {0.0}** | **1** | **(100.0) {0.4}** |
| **2** | **29** | **(78.4) {15.0}** | **8** | **(21.6) {21.6}** | **37** | **(100.0) {16.1}** |
| **3** | **1** | **(100.0) {0.5}** | **0** | **(0.0) {0.0}** | **1** | **(100.0) {0.4}** |
| **7** | **4** | **(80.0) {2.1}** | **1** | **(20.0) {2.7}** | **5** | **(100.0) {2.2}** |
| **8** | **75** | **(94.9) {38.9}** | **4** | **(5.1) {10.8}** | **79** | **(100.0) {34.3}** |
| **10** | **3** | **(75.0) {1.6}** | **1** | **(25.0) {2.7}** | **4** | **(100.0) {1.7}** |
| **12** | **19** | **(63.3) {9.8}** | **11** | **(36.7) {29.7}** | **30** | **(100.0) {13.0}** |
| **15** | **2** | **(100.0) {1.0}** | **0** | **(0.0) {0.0}** | **2** | **(100.0) {0.9}** |
| **16** | **1** | **(100.0) {0.5}** | **0** | **(0.0) {0.0}** | **1** | **(100.0) {0.4}** |
| **18** | **0** | **(0.0) {0.0}** | **1** | **(100.0) {2.7}** | **1** | **(100.0) {0.4}** |
| **22** | **46** | **(85.2) {23.8}** | **8** | **(14.8) {21.6}** | **54** | **(100.0) {23.5}** |
| **24** | **1** | **(33.3) {0.5}** | **2** | **(66.7) {5.4}** | **3** | **(100.0) {1.3}** |
| **26** | **3** | **(75.0) {1.6}** | **1** | **(25.0) {2.7}** | **4** | **(100.0) {1.7}** |
| **28** | **1** | **(100.0) {0.5}** | **0** | **(0.0) {0.0}** | **1** | **(100.0) {0.4}** |
| **29** | **3** | **(100.0) {1.6}** | **0** | **(0.0) {0.0}** | **3** | **(100.0) {1.3}** |
| **36** | **1** | **(100.0) {0.5}** | **0** | **(0.0) {0.0}** | **1** | **(100.0) {0.4}** |
| **43** | **1** | **(100.0) {0.5}** | **0** | **(0.0) {0.0}** | **1** | **(100.0) {0.4}** |
| **44** | **1** | **(100.0) {0.5}** | **0** | **(0.0) {0.0}** | **1** | **(100.0) {0.4}** |
| **45** | **1** | **(100.0) {0.5}** | **0** | **(0.0) {0.0}** | **1** | **(100.0) {0.4}** |
| **Total** | **193** | **(83.9) {100.0}** | **37** | **(16.1) {100.0}** | **230** |  |
| **Percents: (Row) {Col}     Chi^2^= 31.350 df(18) p= 0.0262    Cells_expected<5:_ 31 (82 pct.)** | | | | | | |

**. tables risk cerclage /t /o /c /r**

**Select: (mult = 0)**

| **Outcome:risk** | | | | | | |
| --- | --- | --- | --- | --- | --- | --- |
| **cerclage** | **1** | **%** | **0** | **%** | **Total** | **%** |
| **1** | **5** | **(35.7) {0.9}** | **9** | **(64.3) {0.8}** | **14** | **(100.0) {0.8}** |
| **0** | **569** | **(33.0) {99.1}** | **1153** | **(67.0) {99.2}** | **1722** | **(100.0) {99.2}** |
| **Total** | **574** | **(33.1) {100.0}** | **1162** | **(66.9) {100.0}** | **1736** |  |
| **Percents: (Row) {Col}  Exposure: cerclage = 1 Outcome: risk = 1    Chi^2^= 0.045 df(1) p= 0.8324    Cells_expected<5:_ 1 (25 pct.)   Odds Ratio = 1.13 (95% CI: 0.38-3.37)    (Robins,Greenland,Breslow CI)** | | | | | | |

**. tables risk cortico /t /o /c /r**

**Select: (mult = 0)**

| **Outcome:risk** | | | | | | |
| --- | --- | --- | --- | --- | --- | --- |
| **Corticotherapie** | **0** | **%** | **1** | **%** | **Total** | **%** |
| **0** | **1047** | **(85.3) {90.3}** | **180** | **(14.7) {31.4}** | **1227** | **(100.0) {70.8}** |
| **1** | **110** | **(22.0) {9.5}** | **391** | **(78.0) {68.1}** | **501** | **(100.0) {28.9}** |
| **2** | **3** | **(50.0) {0.3}** | **3** | **(50.0) {0.5}** | **6** | **(100.0) {0.3}** |
| **Total** | **1160** | **(66.9) {100.0}** | **574** | **(33.1) {100.0}** | **1734** |  |
| **Percents: (Row) {Col}     Chi^2^=645.968 df(2) p= 0.0000    Cells_expected<5:_ 2 (33 pct.)** | | | | | | |

**. tables risk cortic2 /t /o /c /r**

**Select: (mult = 0)**

| **Outcome:risk** | | | | | | |
| --- | --- | --- | --- | --- | --- | --- |
| **cortic2** | **1** | **%** | **0** | **%** | **Total** | **%** |
| **1** | **394** | **(77.7) {68.6}** | **113** | **(22.3) {9.7}** | **507** | **(100.0) {29.2}** |
| **0** | **180** | **(14.7) {31.4}** | **1047** | **(85.3) {90.3}** | **1227** | **(100.0) {70.8}** |
| **Total** | **574** | **(33.1) {100.0}** | **1160** | **(66.9) {100.0}** | **1734** |  |
| **Percents: (Row) {Col}  Exposure: cortic2 = 1 Outcome: risk = 1    Chi^2^=643.863 df(1) p= 0.0000    Odds Ratio = 20.28 (95% CI: 15.60-26.36)    (Robins,Greenland,Breslow CI)** | | | | | | |

**. tables risk htagrav /t /o /c /r**

**Select: (mult = 0)**

| **Outcome:risk** | | | | | | |
| --- | --- | --- | --- | --- | --- | --- |
| **HTA gravidique** | **1** | **%** | **0** | **%** | **Total** | **%** |
| **1** | **502** | **(32.9) {87.5}** | **1023** | **(67.1) {88.0}** | **1525** | **(100.0) {87.8}** |
| **0** | **72** | **(34.1) {12.5}** | **139** | **(65.9) {12.0}** | **211** | **(100.0) {12.2}** |
| **Total** | **574** | **(33.1) {100.0}** | **1162** | **(66.9) {100.0}** | **1736** |  |
| **Percents: (Row) {Col}  Exposure: HTA gravidique = 1 Outcome: risk = 1    Chi^2^= 0.122 df(1) p= 0.7273    Odds Ratio = 0.95 (95% CI: 0.70-1.28)    (Robins,Greenland,Breslow CI)** | | | | | | |

**. tables risk preec /t /o /c /r**

**Select: (mult = 0)**

| **Outcome:risk** | | | | | | |
| --- | --- | --- | --- | --- | --- | --- |
| **Preeclampsie** | **0** | **%** | **1** | **%** | **Total** | **%** |
| **1** | **1162** | **(66.9) {100.0}** | **574** | **(33.1) {100.0}** | **1736** | **(100.0) {100.0}** |
| **Total** | **1162** | **(66.9) {100.0}** | **574** | **(33.1) {100.0}** | **1736** |  |
| **Percents: (Row) {Col}  Stratum non-informative** | | | | | | |

**. tables risk htachro /t /o /c /r**

**Select: (mult = 0)**

| **Outcome:risk** | | | | | | |
| --- | --- | --- | --- | --- | --- | --- |
| **HTA chronique** | **1** | **%** | **0** | **%** | **Total** | **%** |
| **1** | **73** | **(41.0) {12.7}** | **105** | **(59.0) {9.0}** | **178** | **(100.0) {10.3}** |
| **0** | **501** | **(32.2) {87.3}** | **1057** | **(67.8) {91.0}** | **1558** | **(100.0) {89.7}** |
| **Total** | **574** | **(33.1) {100.0}** | **1162** | **(66.9) {100.0}** | **1736** |  |
| **Percents: (Row) {Col}  Exposure: HTA chronique = 1 Outcome: risk = 1    Chi^2^= 5.659 df(1) p= 0.0174    Odds Ratio = 1.47 (95% CI: 1.07-2.01)    (Robins,Greenland,Breslow CI)** | | | | | | |

**. tables risk ecl /t /o /c /r**

**Select: (mult = 0)**

| **Outcome:risk** | | | | | | |
| --- | --- | --- | --- | --- | --- | --- |
| **Eclampsie** | **1** | **%** | **0** | **%** | **Total** | **%** |
| **OUI** | **15** | **(31.9) {2.6}** | **32** | **(68.1) {2.8}** | **47** | **(100.0) {2.7}** |
| **NON** | **559** | **(33.1) {97.4}** | **1129** | **(66.9) {97.2}** | **1688** | **(100.0) {97.3}** |
| **Total** | **574** | **(33.1) {100.0}** | **1161** | **(66.9) {100.0}** | **1735** |  |
| **Percents: (Row) {Col}  Exposure: Eclampsie = OUI Outcome: risk = 1    Chi^2^= 0.030 df(1) p= 0.8629    Odds Ratio = 0.95 (95% CI: 0.51-1.76)    (Robins,Greenland,Breslow CI)** | | | | | | |

**. tables risk pvt  /t /o /c /r**

**Select: (mult = 0)**

| **Outcome:risk** | | | | | | |
| --- | --- | --- | --- | --- | --- | --- |
| **PVt** | **0** | **%** | **1** | **%** | **Total** | **%** |
| **0** | **866** | **(67.2) {80.0}** | **422** | **(32.8) {87.0}** | **1288** | **(100.0) {82.2}** |
| **1** | **150** | **(76.9) {13.9}** | **45** | **(23.1) {9.3}** | **195** | **(100.0) {12.4}** |
| **2** | **4** | **(100.0) {0.4}** | **0** | **(0.0) {0.0}** | **4** | **(100.0) {0.3}** |
| **3** | **2** | **(66.7) {0.2}** | **1** | **(33.3) {0.2}** | **3** | **(100.0) {0.2}** |
| **4** | **1** | **(100.0) {0.1}** | **0** | **(0.0) {0.0}** | **1** | **(100.0) {0.1}** |
| **6** | **47** | **(79.7) {4.3}** | **12** | **(20.3) {2.5}** | **59** | **(100.0) {3.8}** |
| **7** | **0** | **(0.0) {0.0}** | **1** | **(100.0) {0.2}** | **1** | **(100.0) {0.1}** |
| **8** | **4** | **(66.7) {0.4}** | **2** | **(33.3) {0.4}** | **6** | **(100.0) {0.4}** |
| **9** | **8** | **(80.0) {0.7}** | **2** | **(20.0) {0.4}** | **10** | **(100.0) {0.6}** |
| **Total** | **1082** | **(69.0) {100.0}** | **485** | **(31.0) {100.0}** | **1567** |  |
| **Percents: (Row) {Col}     Chi^2^= 15.804 df(8) p= 0.0453    Cells_expected<5:_ 11 (61 pct.)** | | | | | | |

**. tables risk ecbut /t /o /c /r**

**Select: (mult = 0)**

| **Outcome:risk** | | | | | | |
| --- | --- | --- | --- | --- | --- | --- |
| **ECBUt** | **0** | **%** | **1** | **%** | **Total** | **%** |
| **0** | **1009** | **(68.6) {94.7}** | **462** | **(31.4) {94.3}** | **1471** | **(100.0) {94.5}** |
| **1** | **19** | **(82.6) {1.8}** | **4** | **(17.4) {0.8}** | **23** | **(100.0) {1.5}** |
| **2** | **2** | **(50.0) {0.2}** | **2** | **(50.0) {0.4}** | **4** | **(100.0) {0.3}** |
| **3** | **28** | **(65.1) {2.6}** | **15** | **(34.9) {3.1}** | **43** | **(100.0) {2.8}** |
| **4** | **2** | **(40.0) {0.2}** | **3** | **(60.0) {0.6}** | **5** | **(100.0) {0.3}** |
| **6** | **0** | **(0.0) {0.0}** | **1** | **(100.0) {0.2}** | **1** | **(100.0) {0.1}** |
| **7** | **3** | **(60.0) {0.3}** | **2** | **(40.0) {0.4}** | **5** | **(100.0) {0.3}** |
| **8** | **2** | **(66.7) {0.2}** | **1** | **(33.3) {0.2}** | **3** | **(100.0) {0.2}** |
| **9** | **1** | **(100.0) {0.1}** | **0** | **(0.0) {0.0}** | **1** | **(100.0) {0.1}** |
| **Total** | **1066** | **(68.5) {100.0}** | **490** | **(31.5) {100.0}** | **1556** |  |
| **Percents: (Row) {Col}     Chi^2^= 7.680 df(8) p= 0.4653    Cells_expected<5:_ 12 (67 pct.)** | | | | | | |

**. tables risk amnio**

**Select: (mult = 0)**

| **risk** | | | |
| --- | --- | --- | --- |
| **amniocentŠse** | **0** | **1** | **Total** |
| **NON** | **1046** | **502** | **1548** |
| **OUI** | **96** | **64** | **160** |
| **REFUS** | **20** | **8** | **28** |
| **Total** | **1162** | **574** | **1736** |

**. select amnio = 1**

**((amnio = 1) and (mult = 0))**

**. tables risk motamnio  /t /o /c /r**

**Select: ((amnio = 1) and (mult = 0))**

| **Outcome:risk** | | | | | | |
| --- | --- | --- | --- | --- | --- | --- |
| **Motif amnio** | **0** | **%** | **1** | **%** | **Total** | **%** |
| **1** | **57** | **(68.7) {59.4}** | **26** | **(31.3) {40.6}** | **83** | **(100.0) {51.9}** |
| **2** | **18** | **(54.5) {18.8}** | **15** | **(45.5) {23.4}** | **33** | **(100.0) {20.6}** |
| **3** | **5** | **(55.6) {5.2}** | **4** | **(44.4) {6.3}** | **9** | **(100.0) {5.6}** |
| **4** | **1** | **(100.0) {1.0}** | **0** | **(0.0) {0.0}** | **1** | **(100.0) {0.6}** |
| **7** | **2** | **(100.0) {2.1}** | **0** | **(0.0) {0.0}** | **2** | **(100.0) {1.3}** |
| **8** | **5** | **(23.8) {5.2}** | **16** | **(76.2) {25.0}** | **21** | **(100.0) {13.1}** |
| **9** | **2** | **(100.0) {2.1}** | **0** | **(0.0) {0.0}** | **2** | **(100.0) {1.3}** |
| **DIAG/GENETIQUE** | **1** | **(100.0) {1.0}** | **0** | **(0.0) {0.0}** | **1** | **(100.0) {0.6}** |
| **PRECAUTION/ATCD** | **2** | **(50.0) {2.1}** | **2** | **(50.0) {3.1}** | **4** | **(100.0) {2.5}** |
| **ACE/ALFAFOETO** | **1** | **(50.0) {1.0}** | **1** | **(50.0) {1.6}** | **2** | **(100.0) {1.3}** |
| **HYDRAMNIOS** | **2** | **(100.0) {2.1}** | **0** | **(0.0) {0.0}** | **2** | **(100.0) {1.3}** |
| **Total** | **96** | **(60.0) {100.0}** | **64** | **(40.0) {100.0}** | **160** |  |
| **Percents: (Row) {Col}     Chi^2^= 20.129 df(10) p= 0.0281    Cells_expected<5:_ 15 (68 pct.)** | | | | | | |

**. select**

**. select mult = 0**

**. select deces <> 1**

**((deces <> 1) and (mult = 0))**

**. tables risk acc /t /o /c /r**

**Select: ((deces <> 1) and (mult = 0))**

| **Outcome:risk** | | | | | | |
| --- | --- | --- | --- | --- | --- | --- |
| **Mode d'accouchement** | **0** | **%** | **1** | **%** | **Total** | **%** |
| **VOIEBASSE** | **540** | **(97.1) {47.1}** | **16** | **(2.9) {3.1}** | **556** | **(100.0) {33.5}** |
| **CESAR** | **508** | **(50.7) {44.3}** | **494** | **(49.3) {96.7}** | **1002** | **(100.0) {60.4}** |
| **VENTOUSE** | **74** | **(100.0) {6.5}** | **0** | **(0.0) {0.0}** | **74** | **(100.0) {4.5}** |
| **FORCEPS** | **8** | **(100.0) {0.7}** | **0** | **(0.0) {0.0}** | **8** | **(100.0) {0.5}** |
| **SIEGE** | **2** | **(100.0) {0.2}** | **0** | **(0.0) {0.0}** | **2** | **(100.0) {0.1}** |
| **ENROUTE** | **2** | **(66.7) {0.2}** | **1** | **(33.3) {0.2}** | **3** | **(100.0) {0.2}** |
| **SPATULES** | **13** | **(100.0) {1.1}** | **0** | **(0.0) {0.0}** | **13** | **(100.0) {0.8}** |
| **Total** | **1147** | **(69.2) {100.0}** | **511** | **(30.8) {100.0}** | **1658** |  |
| **Percents: (Row) {Col}     Chi^2^=407.343 df(6) p= 0.0000    Cells_expected<5:_ 6 (43 pct.)** | | | | | | |

**. tables risk ces /t /o /c /r**

**Select: ((deces <> 1) and (mult = 0))**

| **Outcome:risk** | | | | | | |
| --- | --- | --- | --- | --- | --- | --- |
| **ces** | **1** | **%** | **0** | **%** | **Total** | **%** |
| **1** | **494** | **(49.3) {96.7}** | **508** | **(50.7) {44.3}** | **1002** | **(100.0) {60.4}** |
| **0** | **17** | **(2.6) {3.3}** | **639** | **(97.4) {55.7}** | **656** | **(100.0) {39.6}** |
| **Total** | **511** | **(30.8) {100.0}** | **1147** | **(69.2) {100.0}** | **1658** |  |
| **Percents: (Row) {Col}  Exposure: ces = 1 Outcome: risk = 1    Chi^2^=405.686 df(1) p= 0.0000    Odds Ratio = 36.55 (95% CI: 22.23-60.10)    (Robins,Greenland,Breslow CI)** | | | | | | |

**. tables risk motces /t /o /c /r**

**Select: ((deces <> 1) and (mult = 0))**

| **Outcome:risk** | | | | | | |
| --- | --- | --- | --- | --- | --- | --- |
| **heures Motif cesar** | **0** | **%** | **1** | **%** | **Total** | **%** |
| **1** | **20** | **(100.0) {4.0}** | **0** | **(0.0) {0.0}** | **20** | **(100.0) {2.0}** |
| **2** | **136** | **(72.7) {27.0}** | **51** | **(27.3) {10.3}** | **187** | **(100.0) {18.8}** |
| **3** | **19** | **(67.9) {3.8}** | **9** | **(32.1) {1.8}** | **28** | **(100.0) {2.8}** |
| **4** | **42** | **(93.3) {8.3}** | **3** | **(6.7) {0.6}** | **45** | **(100.0) {4.5}** |
| **5** | **223** | **(35.9) {44.3}** | **399** | **(64.1) {80.8}** | **622** | **(100.0) {62.4}** |
| **6** | **1** | **(100.0) {0.2}** | **0** | **(0.0) {0.0}** | **1** | **(100.0) {0.1}** |
| **7** | **42** | **(100.0) {8.3}** | **0** | **(0.0) {0.0}** | **42** | **(100.0) {4.2}** |
| **8** | **0** | **(0.0) {0.0}** | **1** | **(100.0) {0.2}** | **1** | **(100.0) {0.1}** |
| **PLAC/PRAEVIA** | **3** | **(75.0) {0.6}** | **1** | **(25.0) {0.2}** | **4** | **(100.0) {0.4}** |
| **PROCIDENCE** | **3** | **(100.0) {0.6}** | **0** | **(0.0) {0.0}** | **3** | **(100.0) {0.3}** |
| **INDIC/MAT** | **2** | **(66.7) {0.4}** | **1** | **(33.3) {0.2}** | **3** | **(100.0) {0.3}** |
| **HRP** | **7** | **(21.2) {1.4}** | **26** | **(78.8) {5.3}** | **33** | **(100.0) {3.3}** |
| **SFCHRONIC** | **1** | **(100.0) {0.2}** | **0** | **(0.0) {0.0}** | **1** | **(100.0) {0.1}** |
| **ECHEC/EXTRACø** | **1** | **(100.0) {0.2}** | **0** | **(0.0) {0.0}** | **1** | **(100.0) {0.1}** |
| **HTA/CHRONIQUE** | **1** | **(50.0) {0.2}** | **1** | **(50.0) {0.2}** | **2** | **(100.0) {0.2}** |
| **PRESø/FRONT** | **1** | **(100.0) {0.2}** | **0** | **(0.0) {0.0}** | **1** | **(100.0) {0.1}** |
| **ATCD/PERINEE** | **0** | **(0.0) {0.0}** | **1** | **(100.0) {0.2}** | **1** | **(100.0) {0.1}** |
| **RUPTURE/UTERINE** | **1** | **(50.0) {0.2}** | **1** | **(50.0) {0.2}** | **2** | **(100.0) {0.2}** |
| **Total** | **503** | **(50.5) {100.0}** | **494** | **(49.5) {100.0}** | **997** |  |
| **Percents: (Row) {Col}     Chi^2^=209.017 df(17) p= 0.0000    Cells_expected<5:_ 22 (61 pct.)** | | | | | | |

**. tables risk extrac /t /o /c /r**

**Select: ((deces <> 1) and (mult = 0))**

| **Outcome:risk** | | | | | | |
| --- | --- | --- | --- | --- | --- | --- |
| **extrac** | **1** | **%** | **0** | **%** | **Total** | **%** |
| **1** | **0** | **(0.0) {0.0}** | **95** | **(100.0) {15.0}** | **95** | **(100.0) {14.6}** |
| **0** | **16** | **(2.9) {100.0}** | **540** | **(97.1) {85.0}** | **556** | **(100.0) {85.4}** |
| **Total** | **16** | **(2.5) {100.0}** | **635** | **(97.5) {100.0}** | **651** |  |
| **Percents: (Row) {Col}  Exposure: extrac = 1 Outcome: risk = 1    Chi^2^= 2.803 df(1) p= 0.0941    Cells_expected<5:_ 1 (25 pct.)   Odds Ratio = 0.00 (95% CI: inf- inf)    (Robins,Greenland,Breslow CI)** | | | | | | |

**. tables risk decl /t /o /c /r**

**Select: ((deces <> 1) and (mult = 0))**

| **Outcome:risk** | | | | | | |
| --- | --- | --- | --- | --- | --- | --- |
| **Declenchement** | **0** | **%** | **1** | **%** | **Total** | **%** |
| **NON** | **400** | **(44.9) {34.9}** | **490** | **(55.1) {95.9}** | **890** | **(100.0) {53.7}** |
| **PROSTAGLANDINE** | **384** | **(96.7) {33.5}** | **13** | **(3.3) {2.5}** | **397** | **(100.0) {23.9}** |
| **SYNTO** | **160** | **(96.4) {13.9}** | **6** | **(3.6) {1.2}** | **166** | **(100.0) {10.0}** |
| **PROST+SYNTO** | **126** | **(100.0) {11.0}** | **0** | **(0.0) {0.0}** | **126** | **(100.0) {7.6}** |
| **MYFEGINE** | **30** | **(100.0) {2.6}** | **0** | **(0.0) {0.0}** | **30** | **(100.0) {1.8}** |
| **BALLONNET** | **47** | **(95.9) {4.1}** | **2** | **(4.1) {0.4}** | **49** | **(100.0) {3.0}** |
| **Total** | **1147** | **(69.2) {100.0}** | **511** | **(30.8) {100.0}** | **1658** |  |
| **Percents: (Row) {Col}     Chi^2^=530.022 df(5) p= 0.0000** | | | | | | |

**. tables risk declt /t /o /c /r**

**Select: ((deces <> 1) and (mult = 0))**

| **Outcome:risk** | | | | | | |
| --- | --- | --- | --- | --- | --- | --- |
| **declt** | **1** | **%** | **0** | **%** | **Total** | **%** |
| **1** | **21** | **(2.7) {4.1}** | **747** | **(97.3) {65.1}** | **768** | **(100.0) {46.3}** |
| **0** | **490** | **(55.1) {95.9}** | **400** | **(44.9) {34.9}** | **890** | **(100.0) {53.7}** |
| **Total** | **511** | **(30.8) {100.0}** | **1147** | **(69.2) {100.0}** | **1658** |  |
| **Percents: (Row) {Col}  Exposure: declt = 1 Outcome: risk = 1    Chi^2^=529.318 df(1) p= 0.0000    Odds Ratio = 0.02 (95% CI: 0.01-0.04)    (Robins,Greenland,Breslow CI)** | | | | | | |

**. tables risk motdecl /t /o /c /r**

**Select: ((deces <> 1) and (mult = 0))**

| **Outcome:risk** | | | | | | |
| --- | --- | --- | --- | --- | --- | --- |
| **Motif declenchement** | **0** | **%** | **1** | **%** | **Total** | **%** |
| **1** | **3** | **(100.0) {0.4}** | **0** | **(0.0) {0.0}** | **3** | **(100.0) {0.4}** |
| **2** | **24** | **(100.0) {3.3}** | **0** | **(0.0) {0.0}** | **24** | **(100.0) {3.2}** |
| **3** | **621** | **(97.0) {84.6}** | **19** | **(3.0) {95.0}** | **640** | **(100.0) {84.9}** |
| **4** | **5** | **(100.0) {0.7}** | **0** | **(0.0) {0.0}** | **5** | **(100.0) {0.7}** |
| **5** | **5** | **(100.0) {0.7}** | **0** | **(0.0) {0.0}** | **5** | **(100.0) {0.7}** |
| **6** | **1** | **(100.0) {0.1}** | **0** | **(0.0) {0.0}** | **1** | **(100.0) {0.1}** |
| **7** | **2** | **(100.0) {0.3}** | **0** | **(0.0) {0.0}** | **2** | **(100.0) {0.3}** |
| **9** | **3** | **(100.0) {0.4}** | **0** | **(0.0) {0.0}** | **3** | **(100.0) {0.4}** |
| **RCF/PATHO** | **5** | **(83.3) {0.7}** | **1** | **(16.7) {5.0}** | **6** | **(100.0) {0.8}** |
| **OLIGOAMNIOS** | **1** | **(100.0) {0.1}** | **0** | **(0.0) {0.0}** | **1** | **(100.0) {0.1}** |
| **HTA** | **26** | **(100.0) {3.5}** | **0** | **(0.0) {0.0}** | **26** | **(100.0) {3.4}** |
| **CHOLESTASE/CYTOLYSE** | **4** | **(100.0) {0.5}** | **0** | **(0.0) {0.0}** | **4** | **(100.0) {0.5}** |
| **DIABETE** | **16** | **(100.0) {2.2}** | **0** | **(0.0) {0.0}** | **16** | **(100.0) {2.1}** |
| **RCIU/SFC** | **11** | **(100.0) {1.5}** | **0** | **(0.0) {0.0}** | **11** | **(100.0) {1.5}** |
| **ATCD/CESAR** | **7** | **(100.0) {1.0}** | **0** | **(0.0) {0.0}** | **7** | **(100.0) {0.9}** |
| **Total** | **734** | **(97.3) {100.0}** | **20** | **(2.7) {100.0}** | **754** |  |
| **Percents: (Row) {Col}     Chi^2^= 7.754 df(14) p= 0.9017    Cells_expected<5:_ 22 (73 pct.)** | | | | | | |

**. tables risk mon /t /o /c /r**

**Select: ((deces <> 1) and (mult = 0))**

| **Outcome:risk** | | | | | | |
| --- | --- | --- | --- | --- | --- | --- |
| **Moniteur** | **0** | **%** | **1** | **%** | **Total** | **%** |
| **NORMAL** | **822** | **(74.0) {71.9}** | **289** | **(26.0) {56.6}** | **1111** | **(100.0) {67.1}** |
| **BRADY** | **59** | **(62.1) {5.2}** | **36** | **(37.9) {7.0}** | **95** | **(100.0) {5.7}** |
| **DIP2** | **167** | **(79.9) {14.6}** | **42** | **(20.1) {8.2}** | **209** | **(100.0) {12.6}** |
| **PLAT** | **47** | **(29.9) {4.1}** | **110** | **(70.1) {21.5}** | **157** | **(100.0) {9.5}** |
| **TACHY** | **27** | **(77.1) {2.4}** | **8** | **(22.9) {1.6}** | **35** | **(100.0) {2.1}** |
| **AUTRE** | **0** | **(0.0) {0.0}** | **1** | **(100.0) {0.2}** | **1** | **(100.0) {0.1}** |
| **NONFAIT** | **22** | **(46.8) {1.9}** | **25** | **(53.2) {4.9}** | **47** | **(100.0) {2.8}** |
| **Total** | **1144** | **(69.1) {100.0}** | **511** | **(30.9) {100.0}** | **1655** |  |
| **Percents: (Row) {Col}     Chi^2^=153.111 df(6) p= 0.0000    Cells_expected<5:_ 2 (14 pct.)** | | | | | | |

**. select rcfpat < 9**

**((rcfpat < 9) and ((deces <> 1) and (mult = 0)))**

**. tables risk rcfpat /t /o /c /r**

**Select: ((rcfpat < 9) and ((deces <> 1) and (mult = 0)))**

| **Outcome:risk** | | | | | | |
| --- | --- | --- | --- | --- | --- | --- |
| **rcfpat** | **1** | **%** | **0** | **%** | **Total** | **%** |
| **1** | **197** | **(39.6) {40.5}** | **300** | **(60.4) {26.7}** | **497** | **(100.0) {30.9}** |
| **0** | **289** | **(26.0) {59.5}** | **822** | **(74.0) {73.3}** | **1111** | **(100.0) {69.1}** |
| **Total** | **486** | **(30.2) {100.0}** | **1122** | **(69.8) {100.0}** | **1608** |  |
| **Percents: (Row) {Col}  Exposure: rcfpat = 1 Outcome: risk = 1    Chi^2^= 30.228 df(1) p= 0.0000    Odds Ratio = 1.87 (95% CI: 1.49-2.34)    (Robins,Greenland,Breslow CI)** | | | | | | |

**. tables risk hgie /t /o /c /r**

**Select: ((rcfpat < 9) and ((deces <> 1) and (mult = 0)))**

| **Outcome:risk** | | | | | | |
| --- | --- | --- | --- | --- | --- | --- |
| **H‚morragie d‚livrance** | **0** | **%** | **1** | **%** | **Total** | **%** |
| **NON** | **913** | **(71.2) {94.8}** | **370** | **(28.8) {98.4}** | **1283** | **(100.0) {95.8}** |
| **SYNTO/RU** | **25** | **(89.3) {2.6}** | **3** | **(10.7) {0.8}** | **28** | **(100.0) {2.1}** |
| **PROSTAGL** | **18** | **(90.0) {1.9}** | **2** | **(10.0) {0.5}** | **20** | **(100.0) {1.5}** |
| **EMBOL** | **6** | **(85.7) {0.6}** | **1** | **(14.3) {0.3}** | **7** | **(100.0) {0.5}** |
| **HYSTERECT** | **1** | **(100.0) {0.1}** | **0** | **(0.0) {0.0}** | **1** | **(100.0) {0.1}** |
| **Total** | **963** | **(71.9) {100.0}** | **376** | **(28.1) {100.0}** | **1339** |  |
| **Percents: (Row) {Col}     Chi^2^= 8.834 df(4) p= 0.0654    Cells_expected<5:_ 3 (30 pct.)** | | | | | | |

**. tables risk hgie2 /t /o /c /r**

**Select: ((rcfpat < 9) and ((deces <> 1) and (mult = 0)))**

| **Outcome:risk** | | | | | | |
| --- | --- | --- | --- | --- | --- | --- |
| **hgie2** | **1** | **%** | **0** | **%** | **Total** | **%** |
| **1** | **6** | **(10.7) {1.6}** | **50** | **(89.3) {5.2}** | **56** | **(100.0) {4.2}** |
| **0** | **370** | **(28.8) {98.4}** | **913** | **(71.2) {94.8}** | **1283** | **(100.0) {95.8}** |
| **Total** | **376** | **(28.1) {100.0}** | **963** | **(71.9) {100.0}** | **1339** |  |
| **Percents: (Row) {Col}  Exposure: hgie2 = 1 Outcome: risk = 1    Chi^2^= 8.728 df(1) p= 0.0031    Odds Ratio = 0.30 (95% CI: 0.13-0.70)    (Robins,Greenland,Breslow CI)** | | | | | | |

**. tables risk hgig /t /o /c /r**

**Select: ((rcfpat < 9) and ((deces <> 1) and (mult = 0)))**

| **Outcome:risk** | | | | | | |
| --- | --- | --- | --- | --- | --- | --- |
| **hgig** | **1** | **%** | **0** | **%** | **Total** | **%** |
| **1** | **1** | **(12.5) {0.3}** | **7** | **(87.5) {0.8}** | **8** | **(100.0) {0.6}** |
| **0** | **370** | **(28.8) {99.7}** | **913** | **(71.2) {99.2}** | **1283** | **(100.0) {99.4}** |
| **Total** | **371** | **(28.7) {100.0}** | **920** | **(71.3) {100.0}** | **1291** |  |
| **Percents: (Row) {Col}  Exposure: hgig = 1 Outcome: risk = 1    Chi^2^= 1.036 df(1) p= 0.3087    Cells_expected<5:_ 1 (25 pct.)   Odds Ratio = 0.35 (95% CI: 0.04-2.88)    (Robins,Greenland,Breslow CI)** | | | | | | |

**. select**

**. select mult = 0**

**. tables risk dcfoet /t /o /c /r**

**Select: (mult = 0)**

| **Outcome:risk** | | | | | | |
| --- | --- | --- | --- | --- | --- | --- |
| **dcfoet** | **1** | **%** | **0** | **%** | **Total** | **%** |
| **1** | **63** | **(80.8) {11.0}** | **15** | **(19.2) {1.3}** | **78** | **(100.0) {4.5}** |
| **0** | **511** | **(30.8) {89.0}** | **1147** | **(69.2) {98.7}** | **1658** | **(100.0) {95.5}** |
| **Total** | **574** | **(33.1) {100.0}** | **1162** | **(66.9) {100.0}** | **1736** |  |
| **Percents: (Row) {Col}  Exposure: dcfoet = 1 Outcome: risk = 1    Chi^2^= 83.978 df(1) p= 0.0000    Odds Ratio = 9.43 (95% CI: 5.32-16.71)    (Robins,Greenland,Breslow CI)** | | | | | | |

**. tables risk miu /t /o /c /r**

**Select: (mult = 0)**

| **Outcome:risk** | | | | | | |
| --- | --- | --- | --- | --- | --- | --- |
| **miu** | **1** | **%** | **0** | **%** | **Total** | **%** |
| **1** | **39** | **(73.6) {6.8}** | **14** | **(26.4) {1.2}** | **53** | **(100.0) {3.1}** |
| **0** | **535** | **(31.8) {93.2}** | **1148** | **(68.2) {98.8}** | **1683** | **(100.0) {96.9}** |
| **Total** | **574** | **(33.1) {100.0}** | **1162** | **(66.9) {100.0}** | **1736** |  |
| **Percents: (Row) {Col}  Exposure: miu = 1 Outcome: risk = 1    Chi^2^= 40.557 df(1) p= 0.0000    Odds Ratio = 5.98 (95% CI: 3.22-11.10)    (Robins,Greenland,Breslow CI)** | | | | | | |

**. tables risk img /t /o /c /r**

**Select: (mult = 0)**

| **Outcome:risk** | | | | | | |
| --- | --- | --- | --- | --- | --- | --- |
| **img** | **1** | **%** | **0** | **%** | **Total** | **%** |
| **1** | **22** | **(95.7) {3.8}** | **1** | **(4.3) {0.1}** | **23** | **(100.0) {1.3}** |
| **0** | **552** | **(32.2) {96.2}** | **1161** | **(67.8) {99.9}** | **1713** | **(100.0) {98.7}** |
| **Total** | **574** | **(33.1) {100.0}** | **1162** | **(66.9) {100.0}** | **1736** |  |
| **Percents: (Row) {Col}  Exposure: img = 1 Outcome: risk = 1    Chi^2^= 41.255 df(1) p= 0.0000    Odds Ratio = 46.27 (95% CI: 6.22-344.17)    (Robins,Greenland,Breslow CI)** | | | | | | |

**. tables risk morperi /t /o /c /r**

**Select: (mult = 0)**

| **Outcome:risk** | | | | | | |
| --- | --- | --- | --- | --- | --- | --- |
| **morperi** | **1** | **%** | **0** | **%** | **Total** | **%** |
| **1** | **82** | **(84.5) {14.3}** | **15** | **(15.5) {1.3}** | **97** | **(100.0) {5.6}** |
| **0** | **492** | **(30.0) {85.7}** | **1147** | **(70.0) {98.7}** | **1639** | **(100.0) {94.4}** |
| **Total** | **574** | **(33.1) {100.0}** | **1162** | **(66.9) {100.0}** | **1736** |  |
| **Percents: (Row) {Col}  Exposure: morperi = 1 Outcome: risk = 1    Chi^2^=122.987 df(1) p= 0.0000    Odds Ratio = 12.74 (95% CI: 7.28-22.32)    (Robins,Greenland,Breslow CI)** | | | | | | |

**. tables risk dcprecos /t /o /c /r**

**Select: (mult = 0)**

| **Outcome:risk** | | | | | | |
| --- | --- | --- | --- | --- | --- | --- |
| **dcprecos** | **1** | **%** | **0** | **%** | **Total** | **%** |
| **1** | **40** | **(100.0) {7.8}** | **0** | **(0.0) {0.0}** | **40** | **(100.0) {2.4}** |
| **0** | **472** | **(29.2) {92.2}** | **1147** | **(70.8) {100.0}** | **1619** | **(100.0) {97.6}** |
| **Total** | **512** | **(30.9) {100.0}** | **1147** | **(69.1) {100.0}** | **1659** |  |
| **Percents: (Row) {Col}  Exposure: dcprecos = 1 Outcome: risk = 1    Chi^2^= 91.823 df(1) p= 0.0000    Odds Ratio = -inf. (95% CI: inf- inf)    (Robins,Greenland,Breslow CI)** | | | | | | |

**. tables risk dcinf /t /o /c /r**

**Select: (mult = 0)**

| **Outcome:risk** | | | | | | |
| --- | --- | --- | --- | --- | --- | --- |
| **dcinf** | **1** | **%** | **0** | **%** | **Total** | **%** |
| **1** | **45** | **(100.0) {8.8}** | **0** | **(0.0) {0.0}** | **45** | **(100.0) {2.7}** |
| **0** | **467** | **(28.9) {91.2}** | **1147** | **(71.1) {100.0}** | **1614** | **(100.0) {97.3}** |
| **Total** | **512** | **(30.9) {100.0}** | **1147** | **(69.1) {100.0}** | **1659** |  |
| **Percents: (Row) {Col}  Exposure: dcinf = 1 Outcome: risk = 1    Chi^2^=103.621 df(1) p= 0.0000    Odds Ratio = -inf. (95% CI: inf- inf)    (Robins,Greenland,Breslow CI)** | | | | | | |

**. select deces <> 1**

**((deces <> 1) and (mult = 0))**

**. tables risk sexe /t /o /c /r**

**Select: ((deces <> 1) and (mult = 0))**

| **Outcome:risk** | | | | | | |
| --- | --- | --- | --- | --- | --- | --- |
| **SEXE** | **1** | **%** | **0** | **%** | **Total** | **%** |
| **FEMININ** | **277** | **(31.8) {54.2}** | **595** | **(68.2) {51.9}** | **872** | **(100.0) {52.6}** |
| **MASCULIN** | **234** | **(29.8) {45.8}** | **552** | **(70.2) {48.1}** | **786** | **(100.0) {47.4}** |
| **Total** | **511** | **(30.8) {100.0}** | **1147** | **(69.2) {100.0}** | **1658** |  |
| **Percents: (Row) {Col}  Exposure: SEXE = FEMININ Outcome: risk = 1    Chi^2^= 0.772 df(1) p= 0.3797    Odds Ratio = 1.10 (95% CI: 0.89-1.35)    (Robins,Greenland,Breslow CI)** | | | | | | |

**. tables risk badap /t /o /c /r**

**Select: ((deces <> 1) and (mult = 0))**

| **Outcome:risk** | | | | | | |
| --- | --- | --- | --- | --- | --- | --- |
| **badap** | **1** | **%** | **0** | **%** | **Total** | **%** |
| **1** | **151** | **(55.3) {29.9}** | **122** | **(44.7) {10.7}** | **273** | **(100.0) {16.5}** |
| **0** | **354** | **(25.7) {70.1}** | **1023** | **(74.3) {89.3}** | **1377** | **(100.0) {83.5}** |
| **Total** | **505** | **(30.6) {100.0}** | **1145** | **(69.4) {100.0}** | **1650** |  |
| **Percents: (Row) {Col}  Exposure: badap = 1 Outcome: risk = 1    Chi^2^= 94.008 df(1) p= 0.0000    Odds Ratio = 3.58 (95% CI: 2.74-4.67)    (Robins,Greenland,Breslow CI)** | | | | | | |

**. means pn risk /t**

**Select: ((deces <> 1) and (mult = 0))**

**Syntax: Means PN /BY= risk**

| **h Poids naissance** | | | | | | | | | |
| --- | --- | --- | --- | --- | --- | --- | --- | --- | --- |
| **risk** | **Obs.** | **Sum** | **Mean** | **Variance** | **Std Dev** | **( 95% CI** | **mean )** | **Std Err** |  |
| **0** | **1146** | **3015854.0** | **2631.64** | **366060.0** | **605.03** | **2596.57** | **2666.70** | **17.87** |  |
| **1** | **511** | **649308.0** | **1270.66** | **166319.6** | **407.82** | **1235.22** | **1306.11** | **18.04** |  |
|  |  |  |  |  |  |  |  |  |  |
| **risk** | **Minimum** | **p5** | **p10** | **p25** | **Median** | **p75** | **p90** | **p95** | **Max** |
| **0** | **1054.00** | **1710.00** | **1857.00** | **2180.00** | **2595.00** | **3050.00** | **3433.00** | **3640.00** | **4530.00** |
| **1** | **260.00** | **620.00** | **706.00** | **954.00** | **1300.00** | **1575.00** | **1800.00** | **1904.00** | **2748.00** |

| **Source** | **SS** | **df** | **MS** | **F** | **p Value** |
| --- | --- | --- | --- | --- | --- |
| **Between** | **654609859.05** | **1** | **654609859.05** | **2149.73** | **0.00000000** |
| **Within** | **503961761.97** | **1655** | **304508.62** |  |  |
| **Total** | **1158571621.02** | **1656** | **699620.54** |  |  |
| **Bartlett's test for homogeneity of variance  Chi^2^= 97.557 df(1) p= 0.000** | | | | | |

**. tables pn500 risk /t /o /c /r**

**Select: ((deces <> 1) and (mult = 0))**

| **Outcome:pn500** | | | | | | | | | | | | | | | | | | | | | | |
| --- | --- | --- | --- | --- | --- | --- | --- | --- | --- | --- | --- | --- | --- | --- | --- | --- | --- | --- | --- | --- | --- | --- |
| **risk** | **0 - 499** | **%** | **500 - 999** | **%** | **1000 - 1499** | **%** | **1500 - 1999** | **%** | **2000 - 2499** | **%** | **2500 - 2999** | **%** | **3000 - 3499** | **%** | **3500 - 3999** | **%** | **4000 - 4499** | **%** | **4500 - 4999** | **%** | **Total** | **%** |
| **0** | **0** | **(0.0) {0.0}** | **0** | **(0.0) {0.0}** | **16** | **(1.4) {7.0}** | **166** | **(14.5) {53.4}** | **316** | **(27.6) {95.8}** | **329** | **(28.7) {99.7}** | **220** | **(19.2) {100.0}** | **78** | **(6.8) {100.0}** | **20** | **(1.7) {100.0}** | **1** | **(0.1) {100.0}** | **1146** | **(100.0) {69.2}** |
| **1** | **6** | **(1.2) {100.0}** | **133** | **(26.0) {100.0}** | **212** | **(41.5) {93.0}** | **145** | **(28.4) {46.6}** | **14** | **(2.7) {4.2}** | **1** | **(0.2) {0.3}** | **0** | **(0.0) {0.0}** | **0** | **(0.0) {0.0}** | **0** | **(0.0) {0.0}** | **0** | **(0.0) {0.0}** | **511** | **(100.0) {30.8}** |
| **Total** | **6** | **(0.4) {100.0}** | **133** | **(8.0) {100.0}** | **228** | **(13.8) {100.0}** | **311** | **(18.8) {100.0}** | **330** | **(19.9) {100.0}** | **330** | **(19.9) {100.0}** | **220** | **(13.3) {100.0}** | **78** | **(4.7) {100.0}** | **20** | **(1.2) {100.0}** | **1** | **(0.1) {100.0}** | **1657** |  |
| **Percents: (Row) {Col}     Chi^2^=1156.844 df(9) p= 0.0000    Cells_expected<5:_ 4 (20 pct.)** | | | | | | | | | | | | | | | | | | | | | | |

**. tables risk ppn /t /o /c /r**

**Select: ((deces <> 1) and (mult = 0))**

| **Outcome:risk** | | | | | | |
| --- | --- | --- | --- | --- | --- | --- |
| **ppn** | **1** | **%** | **0** | **%** | **Total** | **%** |
| **1** | **510** | **(50.6) {99.8}** | **498** | **(49.4) {43.4}** | **1008** | **(100.0) {60.8}** |
| **0** | **1** | **(0.2) {0.2}** | **649** | **(99.8) {56.6}** | **650** | **(100.0) {39.2}** |
| **Total** | **511** | **(30.8) {100.0}** | **1147** | **(69.2) {100.0}** | **1658** |  |
| **Percents: (Row) {Col}  Exposure: ppn = 1 Outcome: risk = 1    Chi^2^=471.572 df(1) p= 0.0000    Odds Ratio = 664.64 (95% CI: 93.12-4744.00)    (Robins,Greenland,Breslow CI)** | | | | | | |

**. tables risk tppn /t /o /c /r**

**Select: ((deces <> 1) and (mult = 0))**

| **Outcome:risk** | | | | | | |
| --- | --- | --- | --- | --- | --- | --- |
| **tppn** | **1** | **%** | **0** | **%** | **Total** | **%** |
| **1** | **351** | **(95.6) {68.7}** | **16** | **(4.4) {1.4}** | **367** | **(100.0) {22.1}** |
| **0** | **160** | **(12.4) {31.3}** | **1131** | **(87.6) {98.6}** | **1291** | **(100.0) {77.9}** |
| **Total** | **511** | **(30.8) {100.0}** | **1147** | **(69.2) {100.0}** | **1658** |  |
| **Percents: (Row) {Col}  Exposure: tppn = 1 Outcome: risk = 1    Chi^2^=928.812 df(1) p= 0.0000    Odds Ratio = 155.07 (95% CI: 91.49-262.85)    (Robins,Greenland,Breslow CI)** | | | | | | |

**. tables risk macro /t /o /c /r**

**Select: ((deces <> 1) and (mult = 0))**

| **Outcome:risk** | | | | | | |
| --- | --- | --- | --- | --- | --- | --- |
| **macro** | **1** | **%** | **0** | **%** | **Total** | **%** |
| **1** | **0** | **(0.0) {0.0}** | **99** | **(100.0) {8.6}** | **99** | **(100.0) {6.0}** |
| **0** | **511** | **(32.8) {100.0}** | **1048** | **(67.2) {91.4}** | **1559** | **(100.0) {94.0}** |
| **Total** | **511** | **(30.8) {100.0}** | **1147** | **(69.2) {100.0}** | **1658** |  |
| **Percents: (Row) {Col}  Exposure: macro = 1 Outcome: risk = 1    Chi^2^= 46.906 df(1) p= 0.0000    Odds Ratio = 0.00 (95% CI: inf- inf)    (Robins,Greenland,Breslow CI)** | | | | | | |

**. tables risk macroplus /t /o /c /r**

**Select: ((deces <> 1) and (mult = 0))**

| **Outcome:risk** | | | | | | |
| --- | --- | --- | --- | --- | --- | --- |
| **macroplus** | **1** | **%** | **0** | **%** | **Total** | **%** |
| **1** | **0** | **(0.0) {0.0}** | **21** | **(100.0) {1.8}** | **21** | **(100.0) {1.3}** |
| **0** | **511** | **(31.2) {100.0}** | **1126** | **(68.8) {98.2}** | **1637** | **(100.0) {98.7}** |
| **Total** | **511** | **(30.8) {100.0}** | **1147** | **(69.2) {100.0}** | **1658** |  |
| **Percents: (Row) {Col}  Exposure: macroplus = 1 Outcome: risk = 1    Chi^2^= 9.476 df(1) p= 0.0021    Odds Ratio = 0.00 (95% CI: inf- inf)    (Robins,Greenland,Breslow CI)** | | | | | | |

**. tables risk sga /t /o /c /r**

**Select: ((deces <> 1) and (mult = 0))**

| **Outcome:risk** | | | | | | |
| --- | --- | --- | --- | --- | --- | --- |
| **SGA** | **1** | **%** | **0** | **%** | **Total** | **%** |
| **1** | **110** | **(30.2) {21.5}** | **254** | **(69.8) {22.1}** | **364** | **(100.0) {22.0}** |
| **0** | **401** | **(31.0) {78.5}** | **893** | **(69.0) {77.9}** | **1294** | **(100.0) {78.0}** |
| **Total** | **511** | **(30.8) {100.0}** | **1147** | **(69.2) {100.0}** | **1658** |  |
| **Percents: (Row) {Col}  Exposure: SGA = 1 Outcome: risk = 1    Chi^2^= 0.079 df(1) p= 0.7788    Odds Ratio = 0.96 (95% CI: 0.75-1.24)    (Robins,Greenland,Breslow CI)** | | | | | | |

**. tables risk sgar /t /o /c /r**

**Select: ((deces <> 1) and (mult = 0))**

| **Outcome:risk** | | | | | | |
| --- | --- | --- | --- | --- | --- | --- |
| **SGAR** | **1** | **%** | **0** | **%** | **Total** | **%** |
| **1** | **169** | **(37.0) {33.1}** | **288** | **(63.0) {25.1}** | **457** | **(100.0) {27.6}** |
| **0** | **342** | **(28.5) {66.9}** | **859** | **(71.5) {74.9}** | **1201** | **(100.0) {72.4}** |
| **Total** | **511** | **(30.8) {100.0}** | **1147** | **(69.2) {100.0}** | **1658** |  |
| **Percents: (Row) {Col}  Exposure: SGAR = 1 Outcome: risk = 1    Chi^2^= 11.228 df(1) p= 0.0008    Odds Ratio = 1.47 (95% CI: 1.17-1.85)    (Robins,Greenland,Breslow CI)** | | | | | | |

**. tables risk lgar /t /o /c /r**

**Select: ((deces <> 1) and (mult = 0))**

| **Outcome:risk** | | | | | | |
| --- | --- | --- | --- | --- | --- | --- |
| **LGAR** | **1** | **%** | **0** | **%** | **Total** | **%** |
| **1** | **3** | **(3.4) {0.6}** | **85** | **(96.6) {7.4}** | **88** | **(100.0) {5.3}** |
| **0** | **508** | **(32.4) {99.4}** | **1062** | **(67.6) {92.6}** | **1570** | **(100.0) {94.7}** |
| **Total** | **511** | **(30.8) {100.0}** | **1147** | **(69.2) {100.0}** | **1658** |  |
| **Percents: (Row) {Col}  Exposure: LGAR = 1 Outcome: risk = 1    Chi^2^= 32.750 df(1) p= 0.0000    Odds Ratio = 0.07 (95% CI: 0.02-0.23)    (Robins,Greenland,Breslow CI)** | | | | | | |

**. means terme risk /t**

**Select: ((deces <> 1) and (mult = 0))**

**Syntax: Means TERME /BY= risk**

| **Terme** | | | | | | | | | |
| --- | --- | --- | --- | --- | --- | --- | --- | --- | --- |
| **risk** | **Obs.** | **Sum** | **Mean** | **Variance** | **Std Dev** | **( 95% CI** | **mean )** | **Std Err** |  |
| **0** | **1147** | **42394.0** | **36.96** | **3.28** | **1.81** | **36.86** | **37.07** | **0.05** |  |
| **1** | **511** | **15503.0** | **30.34** | **5.47** | **2.34** | **30.14** | **30.54** | **0.10** |  |
|  |  |  |  |  |  |  |  |  |  |
| **risk** | **Minimum** | **p5** | **p10** | **p25** | **Median** | **p75** | **p90** | **p95** | **Max** |
| **0** | **34.00** | **34.00** | **34.00** | **36.00** | **37.00** | **38.00** | **39.00** | **40.00** | **41.00** |
| **1** | **22.00** | **26.00** | **27.00** | **29.00** | **31.00** | **32.00** | **33.00** | **33.00** | **33.00** |

| **Source** | **SS** | **df** | **MS** | **F** | **p Value** |
| --- | --- | --- | --- | --- | --- |
| **Between** | **15502.67** | **1** | **15502.67** | **3919.65** | **0.0** |
| **Within** | **6549.67** | **1656** | **3.96** |  |  |
| **Total** | **22052.33** | **1657** | **13.31** |  |  |
| **Bartlett's test for homogeneity of variance  Chi^2^= 48.826 df(1) p= 0.000** | | | | | |

**. tables term3 risk /t /o /c /r**

**Select: ((deces <> 1) and (mult = 0))**

| **Outcome:term3** | | | | | | | | | | | | | | | | |
| --- | --- | --- | --- | --- | --- | --- | --- | --- | --- | --- | --- | --- | --- | --- | --- | --- |
| **risk** | **21 - 23** | **%** | **24 - 26** | **%** | **27 - 29** | **%** | **30 - 32** | **%** | **33 - 35** | **%** | **36 - 38** | **%** | **39 - 41** | **%** | **Total** | **%** |
| **0** | **0** | **(0.0) {0.0}** | **0** | **(0.0) {0.0}** | **0** | **(0.0) {0.0}** | **0** | **(0.0) {0.0}** | **265** | **(23.1) {71.4}** | **626** | **(54.6) {100.0}** | **256** | **(22.3) {100.0}** | **1147** | **(100.0) {69.2}** |
| **1** | **2** | **(0.4) {100.0}** | **36** | **(7.0) {100.0}** | **130** | **(25.4) {100.0}** | **237** | **(46.4) {100.0}** | **106** | **(20.7) {28.6}** | **0** | **(0.0) {0.0}** | **0** | **(0.0) {0.0}** | **511** | **(100.0) {30.8}** |
| **Total** | **2** | **(0.1) {100.0}** | **36** | **(2.2) {100.0}** | **130** | **(7.8) {100.0}** | **237** | **(14.3) {100.0}** | **371** | **(22.4) {100.0}** | **626** | **(37.8) {100.0}** | **256** | **(15.4) {100.0}** | **1658** |  |
| **Percents: (Row) {Col}     Chi^2^=1302.890 df(6) p= 0.0000    Cells_expected<5:_ 2 (14 pct.)** | | | | | | | | | | | | | | | | |

**. means termtho risk /t**

**Select: ((deces <> 1) and (mult = 0))**

**Syntax: Means TERMTHO /BY= risk**

| **jours termtho Terme obst‚trical** | | | | | | | | | |
| --- | --- | --- | --- | --- | --- | --- | --- | --- | --- |
| **risk** | **Obs.** | **Sum** | **Mean** | **Variance** | **Std Dev** | **( 95% CI** | **mean )** | **Std Err** |  |
| **0** | **350** | **13209.4** | **37.74** | **3.19** | **1.79** | **37.55** | **37.93** | **0.10** |  |
| **1** | **135** | **4188.10** | **31.02** | **5.97** | **2.44** | **30.61** | **31.44** | **0.21** |  |
|  |  |  |  |  |  |  |  |  |  |
| **risk** | **Minimum** | **p5** | **p10** | **p25** | **Median** | **p75** | **p90** | **p95** | **Max** |
| **0** | **33.40** | **34.60** | **35.31** | **36.40** | **37.90** | **39.00** | **40.00** | **40.60** | **42.60** |
| **1** | **21.90** | **26.98** | **27.36** | **29.60** | **31.90** | **32.90** | **33.60** | **33.90** | **36.30** |

| **Source** | **SS** | **df** | **MS** | **F** | **p Value** |
| --- | --- | --- | --- | --- | --- |
| **Between** | **4397.07** | **1** | **4397.07** | **1109.44** | **0.00000000** |
| **Within** | **1914.29** | **483** | **3.96** |  |  |
| **Total** | **6311.36** | **484** | **13.04** |  |  |
| **Bartlett's test for homogeneity of variance  Chi^2^= 20.495 df(1) p= 0.000** | | | | | |

**. means durgest risk /t**

**Select: ((deces <> 1) and (mult = 0))**

**Syntax: Means DURGEST /BY= risk**

| **Durgest** | | | | | | | | | |
| --- | --- | --- | --- | --- | --- | --- | --- | --- | --- |
| **risk** | **Obs.** | **Sum** | **Mean** | **Variance** | **Std Dev** | **( 95% CI** | **mean )** | **Std Err** |  |
| **0** | **350** | **92473.0** | **264.21** | **156.08** | **12.49** | **262.90** | **265.52** | **0.67** |  |
| **1** | **135** | **29317.0** | **217.16** | **292.57** | **17.10** | **214.25** | **220.07** | **1.47** |  |
|  |  |  |  |  |  |  |  |  |  |
| **risk** | **Minimum** | **p5** | **p10** | **p25** | **Median** | **p75** | **p90** | **p95** | **Max** |
| **0** | **234.00** | **242.00** | **247.10** | **255.00** | **265.00** | **273.00** | **280.00** | **284.00** | **298.00** |
| **1** | **153.00** | **188.80** | **191.60** | **207.00** | **223.00** | **230.00** | **235.00** | **237.00** | **254.00** |

| **Source** | **SS** | **df** | **MS** | **F** | **p Value** |
| --- | --- | --- | --- | --- | --- |
| **Between** | **215624.57** | **1** | **215624.57** | **1111.77** | **0.00000000** |
| **Within** | **93676.19** | **483** | **193.95** |  |  |
| **Total** | **309300.76** | **484** | **639.05** |  |  |
| **Bartlett's test for homogeneity of variance  Chi^2^= 20.647 df(1) p= 0.000** | | | | | |

**. tables risk prema /t /o /c /r**

**Select: ((deces <> 1) and (mult = 0))**

| **Outcome:risk** | | | | | | |
| --- | --- | --- | --- | --- | --- | --- |
| **prema** | **1** | **%** | **0** | **%** | **Total** | **%** |
| **1** | **511** | **(52.1) {100.0}** | **469** | **(47.9) {40.9}** | **980** | **(100.0) {59.1}** |
| **0** | **0** | **(0.0) {0.0}** | **678** | **(100.0) {59.1}** | **678** | **(100.0) {40.9}** |
| **Total** | **511** | **(30.8) {100.0}** | **1147** | **(69.2) {100.0}** | **1658** |  |
| **Percents: (Row) {Col}  Exposure: prema = 1 Outcome: risk = 1    Chi^2^=511.029 df(1) p= 0.0000    Odds Ratio = -inf. (95% CI: inf- inf)    (Robins,Greenland,Breslow CI)** | | | | | | |

**. tables risk prema32 /t /o /c /r**

**Select: ((deces <> 1) and (mult = 0))**

| **Outcome:risk** | | | | | | |
| --- | --- | --- | --- | --- | --- | --- |
| **prema32** | **1** | **%** | **0** | **%** | **Total** | **%** |
| **1** | **405** | **(100.0) {79.3}** | **0** | **(0.0) {0.0}** | **405** | **(100.0) {24.4}** |
| **0** | **106** | **(8.5) {20.7}** | **1147** | **(91.5) {100.0}** | **1253** | **(100.0) {75.6}** |
| **Total** | **511** | **(30.8) {100.0}** | **1147** | **(69.2) {100.0}** | **1658** |  |
| **Percents: (Row) {Col}  Exposure: prema32 = 1 Outcome: risk = 1    Chi^2^=1202.904 df(1) p= 0.0000    Odds Ratio = -inf. (95% CI: inf- inf)    (Robins,Greenland,Breslow CI)** | | | | | | |

**. tables risk transf /t /o /c /r**

**Select: ((deces <> 1) and (mult = 0))**

| **Outcome:risk** | | | | | | |
| --- | --- | --- | --- | --- | --- | --- |
| **Transfert NN** | **0** | **%** | **1** | **%** | **Total** | **%** |
| **NON** | **817** | **(98.7) {71.2}** | **11** | **(1.3) {2.2}** | **828** | **(100.0) {49.9}** |
| **PRIMAIRE** | **199** | **(28.5) {17.3}** | **499** | **(71.5) {97.7}** | **698** | **(100.0) {42.1}** |
| **SECONDAIRE** | **32** | **(100.0) {2.8}** | **0** | **(0.0) {0.0}** | **32** | **(100.0) {1.9}** |
| **KANGOUROU** | **99** | **(99.0) {8.6}** | **1** | **(1.0) {0.2}** | **100** | **(100.0) {6.0}** |
| **Total** | **1147** | **(69.2) {100.0}** | **511** | **(30.8) {100.0}** | **1658** |  |
| **Percents: (Row) {Col}     Chi^2^=935.209 df(3) p= 0.0000** | | | | | | |

**. tables risk transfer /t /o /c /r**

**Select: ((deces <> 1) and (mult = 0))**

| **Outcome:risk** | | | | | | |
| --- | --- | --- | --- | --- | --- | --- |
| **transfer** | **1** | **%** | **0** | **%** | **Total** | **%** |
| **1** | **500** | **(60.2) {97.8}** | **330** | **(39.8) {28.8}** | **830** | **(100.0) {50.1}** |
| **0** | **11** | **(1.3) {2.2}** | **817** | **(98.7) {71.2}** | **828** | **(100.0) {49.9}** |
| **Total** | **511** | **(30.8) {100.0}** | **1147** | **(69.2) {100.0}** | **1658** |  |
| **Percents: (Row) {Col}  Exposure: transfer = 1 Outcome: risk = 1    Chi^2^=674.719 df(1) p= 0.0000    Odds Ratio = 112.53 (95% CI: 61.09-207.31)    (Robins,Greenland,Breslow CI)** | | | | | | |

**. logclose**
