# Supplementary material for: Increased BMI has a linear association with late-onset preeclampsia: A population-based study
Source: PLoS One. 2019 Oct 17;14(10):e0223888. doi: 10.1371/journal.pone.0223888 (PMC6797165; doi:10.1371/journal.pone.0223888)
Supplement: S5 File — (DOCX) [file pone.0223888.s005.docx]

Epi Info

[Results Library](file:///C:\\Epi_Info35\\IResults.htm)

| *Current View:* | C:\DataE\logisticPE.rec: | | | | |
| --- | --- | --- | --- | --- | --- |
| *Record Count:* | 75878 | *(Deleted records excluded)* |  | *Date:* | 05/09/2019 12:42:01 |

LOGISTIC eop = ageg bmi5 EOP

[Next Procedure](file:///C:\Epi_Info35\OUT25.htm#Contents1_2)

Unconditional Logistic Regression

| Term | Odds Ratio | 95% | C.I. | Coefficient | S. E. | Z-Statistic | P-Value |
| --- | --- | --- | --- | --- | --- | --- | --- |
| ageg | 1,0350 | 1,0215 | 1,0487 | 0,0344 | 0,0067 | 5,1358 | 0,0000 |
| bmi5 | 1,0394 | 1,0262 | 1,0528 | 0,0386 | 0,0065 | 5,9128 | 0,0000 |
| CONSTANT | * | * | * | -6,7367 | 0,2323 | -29,0022 | 0,0000 |

| Convergence: | Converged |
| --- | --- |
| Iterations: | 5 |
| Final -2*Log-Likelihood: | 5764,3043 |
| Cases included: | 68678 |

| Test | Statistic | D.F. | P-Value |
| --- | --- | --- | --- |
| Score | 69,3515 | 2 | 0,0000 |
| Likelihood Ratio | 65,9837 | 2 | 0,0000 |

LOGISTIC lop = ageg bmi5 LOP

Unconditional Logistic Regression

| Term | Odds Ratio | 95% | C.I. | Coefficient | S. E. | Z-Statistic | P-Value |
| --- | --- | --- | --- | --- | --- | --- | --- |
| ageg | 1,0151 | 1,0061 | 1,0242 | 0,0150 | 0,0045 | 3,2998 | 0,0010 |
| bmi5 | 1,0562 | 1,0476 | 1,0648 | 0,0546 | 0,0042 | 13,0976 | 0,0000 |
| CONSTANT | * | * | * | -5,7970 | 0,1526 | -37,9791 | 0,0000 |

| Convergence: | Converged |
| --- | --- |
| Iterations: | 5 |
| Final -2*Log-Likelihood: | 11088,1422 |
| Cases included: | 69283 |

| Test | Statistic | D.F. | P-Value |
| --- | --- | --- | --- |
| Score | 197,0833 | 2 | 0,0000 |
| Likelihood Ratio | 175,6268 | 2 | 0,0000 |

LOGISTIC PREEC = ageg bmi5 ALL PREECLAMPSIA 

Unconditional Logistic Regression

| Term | Odds Ratio | 95% | C.I. | Coefficient | S. E. | Z-Statistic | P-Value |
| --- | --- | --- | --- | --- | --- | --- | --- |
| ageg | 1,0217 | 1,0144 | 1,0291 | 0,0215 | 0,0037 | 5,8515 | 0,0000 |
| bmi5 | 1,0501 | 1,0430 | 1,0572 | 0,0489 | 0,0035 | 14,1439 | 0,0000 |
| CONSTANT | * | * | * | -5,4192 | 0,1251 | -43,3344 | 0,0000 |

| Convergence: | Converged |
| --- | --- |
| Iterations: | 5 |
| Final -2*Log-Likelihood: | 15707,2580 |
| Cases included: | 71152 |

| Test | Statistic | D.F. | P-Value |
| --- | --- | --- | --- |
| Score | 258,4664 | 2 | 0,0000 |
| Likelihood Ratio | 236,6078 | 2 | 0,0000 |
