# Supplementary material for: Increased BMI has a linear association with late-onset preeclampsia: A population-based study
Source: PLoS One. 2019 Oct 17;14(10):e0223888. doi: 10.1371/journal.pone.0223888 (PMC6797165; doi:10.1371/journal.pone.0223888)
Supplement: S6 File — (DOCX) [file pone.0223888.s006.docx]

**Epi Info**

[Results Library](file:///C:\\Epi_Info35\\IResults.htm)

| *Current View:* | **C:\DataE\logisticPE.rec:** | | | | |
| --- | --- | --- | --- | --- | --- |
| *Record Count:* | **75878** | *(Deleted records excluded)* |  | *Date:* | **03/09/2019 11:45:39** |

**LOGISTIC PREEC = ageg bmi5 DIABETE HTACHRO p1 TABAC**

[Next Procedure](file:///C:\Epi_Info35\OUT24.htm#Contents1_2)

**Unconditional Logistic Regression**

| **Term** | **Odds Ratio** | **95%** | **C.I.** | **Coefficient** | **S. E.** | **Z-Statistic** | **P-Value** |
| --- | --- | --- | --- | --- | --- | --- | --- |
| **ageg** | 1,0394 | 1,0310 | 1,0479 | 0,0387 | 0,0042 | 9,2989 | 0,0000 |
| **bmi5** | 1,0465 | 1,0390 | 1,0541 | 0,0455 | 0,0037 | 12,3560 | 0,0000 |
| **DIABETE** | 1,0815 | 0,9412 | 1,2428 | 0,0784 | 0,0709 | 1,1051 | 0,2691 |
| **HTACHRO** | 5,6255 | 4,6727 | 6,7726 | 1,7273 | 0,0947 | 18,2436 | 0,0000 |
| **p1** | 2,4279 | 2,1721 | 2,7138 | 0,8870 | 0,0568 | 15,6177 | 0,0000 |
| **TABAC** | 0,7615 | 0,6410 | 0,9046 | -0,2725 | 0,0879 | -3,1004 | 0,0019 |
| **CONSTANT** | * | * | * | -6,2323 | 0,1561 | -39,9270 | 0,0000 |

| **Convergence:** | Converged |
| --- | --- |
| **Iterations:** | 8 |
| **Final -2*Log-Likelihood:** | 15180,6964 |
| **Cases included:** | 71039 |

| **Test** | **Statistic** | **D.F.** | **P-Value** |
| --- | --- | --- | --- |
| **Score** | 1145,4974 | 6 | 0,0000 |
| **Likelihood Ratio** | 750,3130 | 6 | 0,0000 |

[Previous Dataset](file:///C:\\Epi_Info35\\OUT24.htm#Results1)[Results Library](file:///C:\Epi_Info35\IResults.htm)

**LOGISTIC eop = ageg bmi5 DIABETE HTACHRO p1 TABAC**

[Next Procedure](file:///C:\\Epi_Info35\\OUT24.htm#Contents2_2)

**Unconditional Logistic Regression**

| **Term** | **Odds Ratio** | **95%** | **C.I.** | **Coefficient** | **S. E.** | **Z-Statistic** | **P-Value** |
| --- | --- | --- | --- | --- | --- | --- | --- |
| **ageg** | 1,0491 | 1,0336 | 1,0647 | 0,0479 | 0,0076 | 6,3267 | 0,0000 |
| **bmi5** | 1,0342 | 1,0202 | 1,0483 | 0,0336 | 0,0069 | 4,8558 | 0,0000 |
| **DIABETE** | 0,7392 | 0,5590 | 0,9776 | -0,3022 | 0,1426 | -2,1189 | 0,0341 |
| **HTACHRO** | 8,1607 | 6,0665 | 10,9777 | 2,0993 | 0,1513 | 13,8753 | 0,0000 |
| **p1** | 2,1712 | 1,7702 | 2,6632 | 0,7753 | 0,1042 | 7,4405 | 0,0000 |
| **TABAC** | 0,8764 | 0,6507 | 1,1804 | -0,1320 | 0,1519 | -0,8685 | 0,3851 |
| **CONSTANT** | * | * | * | -7,3525 | 0,2864 | -25,6759 | 0,0000 |

| **Convergence:** | Converged |
| --- | --- |
| **Iterations:** | 9 |
| **Final -2*Log-Likelihood:** | 5571,3581 |
| **Cases included:** | 68521 |

| **Test** | **Statistic** | **D.F.** | **P-Value** |
| --- | --- | --- | --- |
| **Score** | 525,0047 | 6 | 0,0000 |
| **Likelihood Ratio** | 256,6744 | 6 | 0,0000 |

[Previous Dataset](file:///C:\\Epi_Info35\\OUT24.htm#Results2)[Results Library](file:///C:\Epi_Info35\IResults.htm)

**LOGISTIC lop = ageg bmi5 DIABETE HTACHRO p1 TABAC**

[Next Procedure](file:///C:\\Epi_Info35\\OUT24.htm#Contents3_2)

**Unconditional Logistic Regression**

| **Term** | **Odds Ratio** | **95%** | **C.I.** | **Coefficient** | **S. E.** | **Z-Statistic** | **P-Value** |
| --- | --- | --- | --- | --- | --- | --- | --- |
| **ageg** | 1,0336 | 1,0232 | 1,0441 | 0,0330 | 0,0051 | 6,4235 | 0,0000 |
| **bmi5** | 1,0525 | 1,0434 | 1,0617 | 0,0512 | 0,0044 | 11,5512 | 0,0000 |
| **DIABETE** | 1,1566 | 0,9777 | 1,3683 | 0,1455 | 0,0858 | 1,6964 | 0,0898 |
| **HTACHRO** | 4,9592 | 3,9319 | 6,2548 | 1,6012 | 0,1184 | 13,5210 | 0,0000 |
| **p1** | 2,4477 | 2,1337 | 2,8079 | 0,8951 | 0,0700 | 12,7797 | 0,0000 |
| **TABAC** | 0,7380 | 0,5951 | 0,9151 | -0,3038 | 0,1098 | -2,7681 | 0,0056 |
| **CONSTANT** | * | * | * | -6,6327 | 0,1911 | -34,7079 | 0,0000 |

| **Convergence:** | Converged |
| --- | --- |
| **Iterations:** | 9 |
| **Final -2*Log-Likelihood:** | 10755,3252 |
| **Cases included:** | 69125 |

| **Test** | **Statistic** | **D.F.** | **P-Value** |
| --- | --- | --- | --- |
| **Score** | 730,2434 | 6 | 0,0000 |
| **Likelihood Ratio** | 495,1417 | 6 | 0,0000 |
