# Supplementary material for: Increased BMI has a linear association with late-onset preeclampsia: A population-based study
Source: PLoS One. 2019 Oct 17;14(10):e0223888. doi: 10.1371/journal.pone.0223888 (PMC6797165; doi:10.1371/journal.pone.0223888)
Supplement: Attachment — Submitted filename: Response to Reviewers.docx [file pone.0223888.s007.docx]

**[PONE-D-19-21680] - [EMID:49f54e1ae9a6df0f]**

After comments of the reviewers, we have shortened the title which is now:

**Increased BMI has a linear association with late-onset**

**preeclampsia: a population-based study**

When submitting your revision, we need you to address these additional requirements.

1. Please ensure that your manuscript meets PLOS ONE's style requirements, including those for file naming. The PLOS ONE style templates can be found at

<http://www.journals.plos.org/plosone/s/file?id=wjVg/PLOSOne_formatting_sample_main_body.pdf> and <http://www.journals.plos.org/plosone/s/file?id=ba62/PLOSOne_formatting_sample_title_authors_affiliations.pdf>

Verified

2. We note that you have indicated that data from this study are available upon request. PLOS only allows data to be available upon request if there are legal or ethical restrictions on sharing data publicly. For information on unacceptable data access restrictions, please see <http://journals.plos.org/plosone/s/data-availability#loc-unacceptable-data-access-restrictions>.

In your revised cover letter, please address the following prompts:

a) If there are ethical or legal restrictions on sharing a de-identified data set, please explain them in detail (e.g., data contain potentially identifying or sensitive patient information) and who has imposed them (e.g., an ethics committee). Please also provide contact information for a data access committee, ethics committee, or other institutional body to which data requests may be sent.

b) If there are no restrictions, please upload the minimal anonymized data set necessary to replicate your study findings as either Supporting Information files or to a stable, public repository and provide us with the relevant URLs, DOIs, or accession numbers. Please see <http://www.bmj.com/content/340/bmj.c181.long> for guidelines on how to de-identify and prepare clinical data for publication. For a list of acceptable repositories, please see <http://journals.plos.org/plosone/s/data-availability#loc-recommended-repositories>.

We will update your Data Availability statement on your behalf to reflect the information you provide.

We did not understand at first, we will send all the anonymized calculations

 3. Please include a separate caption for each figure in your manuscript.

Figures will be added separately in TIFF format

4. Please upload a copy of Figure 5, to which you refer in your text on page 8. If the figure is no longer to be included as part of the submission please remove all reference to it within the text.

It was a typing mistake, in fact it is Figure 4, corrected

****************************************************************************

5. Review Comments to the Author

Please use the space provided to explain your answers to the questions above. You may also include additional comments for the author, including concerns about dual publication, research ethics, or publication ethics. (Please upload your review as an attachment if it exceeds 20,000 characters)

**Reviewer #1**: In this paper, authors did a 18 year-observational cohort study to analyse the parameters related with EOP and LOP and compared their different effects on these two preeclampsia. It is a relatively comprehensive research based on so large number of cases. And authors got the interesting findings that increment of 5 years for maternal age rising and incidence of preeclampsia were strictly parallel for EOP and LOP, while increment of 5kg/m² for BMI was only associated with LOP. That can give us some clues on the different mechanism of EOP and LOP.

There are some problems need to be modified:

1, In the method, authors quoted the levels of maternity, like level 1, level 3, but didn't give some background of the definition of the levels for maternity. They need introduce the division of levels.

Sentences have been added page 4 (in blue in the text):

“Level 1 maternities are allowed to manage pregnancies after 35 weeks of gestation, without severe risk factors (preeclampsia, multiple pregnancies, in utero fetal deaths, medical termination of pregnancies, complicated gestational diabetes). Level 2 maternities are allowed to manage deliveries of 33 weeks onward; there are never been level 2 maternities in South-Reunion. Level 3 maternities take care of all pregnancies delivering after 21 weeks gestation and all complicated pregnancies (see level 1 exclusions).

Pregnancies are well followed (an average of 9 prenatal visits and 4 ultrasonographies. The mean gestational age at first (“datation”) ultrasonography being 11 weeks.”

2, In the findings, authors found that increment of 5 (years for age, 5kg/m² for BMI) rising maternal ages and incidence of preeclampsia were strictly parallel for EOP and LOP, while increment of BMI was only associated with LOP. It is hard to understand when put 5kg/m² for BMI with 5 years for age to explain the relationship between maternal ages and incidence of preeclampsia. It would be better if they put the information of 5kg/m² for BMI down when concluded that BMI was only associated with LOP.

Increments by 5 years of age, and by 5 kg/m² indeed look similar on the graphs and therefore may be confusing. We have added in the different figures clearly in abscissa years of ages or kg/m².The statistical comparison using 5-unit intervals (BMI and years of age) allows to evaluate more homogeneous data than continuous variables with lower intergroup variability.

Further, we have added in Table 3 a Logistic bivariate regression analysis between maternal ages and pre-pregnancy BMI showing their respective interactions. We have added, page 7 the sentence:

“Also Table 3 shows the bivariate interaction between maternal ages and BMI both together. Adjusted Odds ratios for maternal ages and BMI are similar in EOP women (1.035 [1.02-1.05], 1.039 [1.03-1.05] respectively. This is not the case for LOP: aOR for maternal ages is 1.015 [1.006-1.024] while it raises at 1.056 [1.05-1.06] for maternal pre-pregnancy BMI. In all preeclamptic women, there is a higher increment for BMI, aOR 1.05 (i.e. an increment of 5% for each increment of 5kg/m²) than for maternal ages aOR 1.03 (an increment of 3% for each increment of 5 years of age). But this effect is concentrated only in LOP. Fig 1-2 and Fig 4 show geometrically that indeed, in one case (pre-pregnancy BMI) the two curves diverge by an angle of ap. 30°, while it is a parallel rise when we consider maternal ages”.

3, Some abbreviations, like LDW, VLDW, GDM, ppBMI need to write their full names when first using them in this manuscript: corrected page 4

And authors should make the whole paper be more organised.

The introduction and the conclusions have been completely re-written (in blue in the revised text)

**Reviewer #2:** Pierre-Yves Robillard et al. investigated the effect of BMI (body mass index) on EOP (early onset preeclampsia) vs LOP (late onset) by a long term observational cohort study. And they found that increment of maternal BMI was only associated with LOP during pregnancy. This was a great job. However, there were some issues in the manuscript.
And the suggestion of the manuscript followed:

1. The authors did not highlight the significance of BMI for preeclampsia such as EOP or LOP in the introduction, and there was some confusions in the logical structure in the part;

The introduction and the conclusions have been completely re-written (in blue in the revised text)

2. The maternal BMI analysis of the cohort study was not enough in the Results. It was suggested to set a time point for maternal BMI before and during pregnancy.

All data are pre-pregnancy BMI. The study focuses on pre-pregnancy BMI only, not on weight gain during pregnancy. We have added in material and methods, page 5 the sentence:

“Pre-pregnancy BMI (ppBMI),was calculated on the recalled pre-pregnancy weight by patients themselves in the majority of cases, controlled by the booking weight at first visit (average 6-8 weeks), always written down in the maternity booklet.”

3. The format of Table 4 needs to be revised to meet the requirements of article publication; Table 1-2 has too many entries and needs to be re-integrated and revised

- Table 1-3 have been deleted and replaced by a synthetic Table which is now Table 1.
- Table 4 (logistic regression analysis), now Table 2 and 3 have been revised to meet the requirements

Figures 1-4 should be illustrated with brief annotations.

We have added in the different figures clearly in abscissa years of ages or kg/m².

5. The limitations of the paper need to be explained in detail;

The paragraph is the last one of page 10 (in blue in the text)

6. The conclusion of the paper did not match the title, so the conclusion should be written around the center of the paper.

THE TITLE has been highly shortened and is now:

**Increased BMI has a linear association with late-onset preeclampsia: a population-based study**

The introduction and the conclusions have been completely re-written (in blue in the revised text)
